# Association analysis and functional annotation of imputed sequence data within genomic regions influencing resistance to gastro-intestinal parasites detected by an LDLA approach in a nucleus flock of Sarda dairy sheep

**DOI:** 10.1186/s12711-021-00690-7

**Published:** 2022-01-03

**Authors:** Sara Casu, Mario Graziano Usai, Tiziana Sechi, Sotero L. Salaris, Sabrina Miari, Giuliana Mulas, Claudia Tamponi, Antonio Varcasia, Antonio Scala, Antonello Carta

**Affiliations:** 1 Genetics and Biotechnology – Agris Sardegna, Olmedo, Italy; 2 Department of Veterinary Medicine, University of Sassari, Sassari, Italy

## Abstract

**Background:**

Gastroinestinal nematodes (GIN) are one of the major health problem in grazing sheep. Although genetic variability of the resistance to GIN has been documented, traditional selection is hampered by the difficulty of recording phenotypes, usually fecal egg count (FEC). To identify causative mutations or markers in linkage disequilibrium (LD) to be used for selection, the detection of quantitative trait loci (QTL) for FEC based on linkage disequilibrium-linkage analysis (LDLA) was performed on 4097 ewes (from 181 sires) all genotyped with the OvineSNP50 Beadchip. Identified QTL regions (QTLR) were imputed from whole-genome sequences of 56 target animals of the population. An association analysis and a functional annotation of imputed polymorphisms in the identified QTLR were performed to pinpoint functional variants with potential impact on candidate genes identified from ontological classification or differentially expressed in previous studies.

**Results:**

After clustering close significant locations, ten QTLR were defined on nine *Ovis aries* chromosomes (OAR) by LDLA. The ratio between the ANOVA estimators of the QTL variance and the total phenotypic variance ranged from 0.0087 to 0.0176. QTL on OAR4, 12, 19, and 20 were the most significant. The combination of association analysis and functional annotation of sequence data did not highlight any putative causative mutations. None of the most significant SNPs showed a functional effect on genes’ transcript. However, in the most significant QTLR, we identified genes that contained polymorphisms with a high or moderate impact, were differentially expressed in previous studies, contributed to enrich the most represented GO process (regulation of immune system process, defense response). Among these, the most likely candidate genes were: *TNFRSF1B* and *SELE* on OAR12, *IL5RA* on OAR19, *IL17A*, *IL17F*, *TRIM26*, *TRIM38*, *TNFRSF21*, *LOC101118999*, *VEGFA*, and *TNF* on OAR20.

**Conclusions:**

This study performed on a large experimental population provides a list of candidate genes and polymorphisms which could be used in further validation studies. The expected advancements in the quality of the annotation of the ovine genome and the use of experimental designs based on sequence data and phenotypes from multiple breeds that show different LD extents and gametic phases may help to identify causative mutations.

**Supplementary Information:**

The online version contains supplementary material available at 10.1186/s12711-021-00690-7.

## Background

Gastrointestinal nematodes (GIN) are one of the major health problems in grazing animals [1]. GIN infections result in important yield reductions and higher production costs due to veterinary treatments and higher culling rates [2]. Moreover, chemical treatments involve the risk of drug residues in the food and environment and the appearance of anthelmintic resistance, that has been reported in several countries [3–6]. In sheep, GIN control strategies may also include management practices such as soil tillage or rotational grazing that aim at reducing pasture contamination [7, 8]. Alternative approaches to limit GIN infection rely on nutritional schemes based on either grazing crops with anthelmintic proprieties, such as chicory (*Cichorium intybus*), sulla (*Hedysarum coronarium*), sainfoin (*Onobrychus viciifolia*) and sericea lespedeza (*Lespedeza cuneata*) [9], or supplementation with tannins and/or proteins; but even these approaches are difficult to apply, especially in extensive or semi-extensive systems.

Fecal egg count (FEC), i.e. the number of parasite eggs per g of faeces, has been largely used as a proxy trait to measure individual resistance to GIN. Selective breeding of animals with enhanced resistance to GIN has been suggested for the sustainable control of parasite infections in sheep since genetic variation between individuals and breeds has been documented. Indeed, estimates of the heritability of proxy traits for GIN resistance in sheep ranges from 0.01 to 0.65 [10], but it is generally moderate for FEC (0.25–0.33 [11]; 0.16 [12]; 0.21–0.55 [13]; and 0.18–0.35 [14]). Thus, breeding for resistance to GIN can be considered in sheep but implies structured selection schemes and accurate recording of both performances and pedigree information, which are essential for genetic evaluation. However, the inclusion of GIN resistance in current breeding schemes is hampered by the difficulty to record FEC on a large scale since its measure is too laborious and costly in field conditions. For this reason, several studies have been carried out to dissect the genetic determinism of GIN resistance with the final aim of setting up breeding schemes that are based on molecular information rather than large-scale recording for progeny testing. Such studies have followed the development of the molecular biology and omic sciences and the concomitant advancement of the statistical methodologies. The first studies were based on sparse maps of molecular markers, such as microsatellites, and used linkage analysis on family-structured populations [15]. In spite of the large number of genomic regions detected in sheep [16–18], low significance levels and the low accuracy of localisations made marker-assisted selection unfeasible. Later on, the development of single nucleotide polymorphism (SNP) arrays with medium and high densities and the application of enhanced statistical methods allowed to extend the analysis at the population level and to increase the power of detection and the accuracy of localisations [19–23]. More recently, the availability of high-throughput sequencing technologies and increasingly accurate genome annotations may allow the discovery of new polymorphisms in DNA or RNA sequences and the classification of their effects on genes that are more and more well-known in terms of functions.

The Sarda breed is the most important Italian dairy sheep breed with around three million heads in approximately 10,000 flocks (Regional Department for Agriculture, unpublished observations). Sheep breeding has traditionally been the most important livestock production in Sardinia. Farming systems vary from semi-extensive to semi-intensive with a wide-spread use of grazing on natural pastures and forage crops where infection from GIN is unavoidable. The most represented nematodes species are *Teladorsagia circuncincta*, *Trichostrongylus *spp., *Haemonchus contortus*, *Teladorsagia trifurcata*, *Cooperia *spp., while *Oesophagostomum venulosum* and *Nematodirus *spp. are found in negligible quantities [24]. The prevalence rate in terms of worm egg count generally increases in the summer-autumn period. In these conditions, most farmers have to administer anthelmintics, often without well planned protocols in terms of individual diagnosis, doses and frequency of treatments. Anthelmintic treatments concern 99.4% of the sheep farms on the island, with on average 1.54 treatments per year that are mainly carried out with benzimidazoles (47.8%), levamisole 21.1%, avermectin (12.7%) and probenzimidazoles (11.5%) [25]. Thus, the control of GIN implies high costs, organizational efforts and further economic losses related to the rules that limit drug residues in milk. In this situation, selective breeding is an attractive option also for Sarda sheep. The current breeding scheme is implemented on about 8% of the purebred population for which yield traits and pedigree data are recorded (Herd Book). The main selection objectives are milk yield per lactation, scrapie resistance, and udder morphology [26]. With the aim of assessing the feasibility of a marker-assisted selection (MAS) scheme for resistance to GIN based on causative mutations or markers in linkage disequilibrium (LD), which does not need large-scale FEC recording, the Regional Agency for Agricultural Research (AGRIS) has established since the late 1990s an experimental population for which the individuals are genotyped with SNP arrays and routinely measured for FEC, as well as other production and functional traits. More recently, a target sample of influential animals from this population was whole-genome re-sequenced and SNP genotypes were imputed to the whole population.

The aim of this study was to identity QTL segregating in the Sarda breed and to search for candidate genes and causative mutations by the functional annotation and association analysis of imputed Sarda sequence data in these target regions.

## Methods

### Experimental population

The nucleus flock of the Sarda breed, that is described in more detail in [26, 27], derives from a backcross population of Sarda $\times$ Lacaune ewes created in 1999 by mating 10 F1 Sarda $\times$ Lacaune rams with purebred Sarda ewes. Thereafter, the generations of ewes that were produced until now, were obtained by mating adult ewes of the nucleus flock exclusively with rams coming from the Sarda Herd Book. This has led to a progressive reduction of the proportion of Lacaune blood in the experimental population, which is negligible in the latest generations (around 0.4%). The average size of the flock is about 900 milked ewes per year with a replacement rate of 25 to 30%. The flock is raised on an experimental farm located in the south of Sardinia that generally shows a semi-arid Mediterranean climate with important variations in rainfall and temperatures across seasons and years. The flock is managed following the traditional farming system adopted on the island, which is based on grazing natural or cultivated swards (mainly ryegrass and berseem clover) and supplements of hay, silage and concentrate. Lambings of most of adult ewes occur in the autumn, and those of the remaining ewes and of the primiparous ewes occur in late winter or early spring. Ewes are usually bred in management groups depending on the lambing period. They are milked twice a day by machine from after lamb separation (one month after lambing) until the early summer period when they are progressively and almost simultaneously dried off.

### Molecular data

All the ewes of the experimental population born from 1999 to 2017 (n = 4355) and their sires (n = 181, including the 10 F1) and 11 Sarda grandsires were genotyped with the OvineSNP50 Beadchip (50 k hereafter). SNP editing was performed using the call rate and minor allele frequency (MAF) thresholds of 95% and 1%, respectively. The ovine genome assembly v4.0 and the SNPchimMpv.3 software [28] were used to construct the genetic map by assuming 1 Mb = 1 cM. Unmapped SNPs and SNPs on sex chromosomes were not included in the study. Finally, 43,390 SNPs were retained for further analyses.

Among the 4547 genotyped animals, 56 had also been fully re-sequenced within the framework of previous projects. The choice of these 56 animals was based on the assumption that they carried opposite alleles for specific QTL segregating in the Sarda breed and identified in our previous investigations [29] or because they had many progeny in the experimental population. The first group (24 animals, including two Sarda rams and 22 daughters of Sarda rams) had been whole-genome re-sequenced with a target coverage of 12X. The other 32 animals were Sarda sires chosen among those with a higher impact on the population, more recently re-sequenced on an Illumina HiSeq 3000 sequencer and a 30X target coverage. Whole-genome sequence (WGS) data was processed with a pipeline implemented with Snakemake [30], and developed at CRS4 (Center For Advanced Studies, Research and Development in Sardinia https://www.crs4.it/)) available at https://github.com/solida-core. Briefly, adapter sequences were removed from the short reads, then low-quality ends were trimmed, and sequences shorter than 25 bp after trimming were removed with the TrimGalore (v0.4.5) software [31]. The quality of the reads, before and after trimming, was evaluated with the Fastqc (v0.11.5) tool [32]. Trimmed reads were aligned to the *Ovis aries* reference genome v4.0 (https://www.ncbi.nlm.nih.gov/assembly/GCF_000298735.2) using the Burrow-Wheeler Aligner (BWA v0.7.15) program [33]. Alignments were further sorted, converted to a CRAM file and indexed with Samtools (v1.6) [34]. PCR duplicates were detected with the Picard (v2.18.9) tool [35]. After alignment, joint single nucleotide variants (SNV) (SNPs and insertion-deletions (INDELs)) calling was performed using the GATK (v4.0.11.0) software [36], according to the GATK Best practices workflow [37]. In order to apply the GATK Variant Quality Score Recalibration, first we ran an initial round of SNP calling and only used the top 5% SNPs with the highest quality scores.

### Phenotypes

FEC was the proxy trait used to assess GIN resistance under natural conditions of infection in the experimental flock. Periodically, a sample of ~ 50 ewes that represented the different management groups, was monitored to evaluate the percentage of infected animals and decide whether to sample the whole flock and possibly administrate anthelmintic treatment. The number of eggs of strongyles per g was determined using a copromicroscopic test according to the McMaster technique [38] on individual samples. When the number of infected animals and the level of infestation were considered sufficient to appreciate individual variability, individual FEC were measured on the whole flock. During the first three years of measurement, coprocultures of pooled samples were also performed at each round of scoring in order to identify GIN genera using the technique and the identification keys of [39, 40]. The results of pooled faecal cultures (mean of 4 cultures and 200 to 400 larval identifications) indicated that *H. contortus*, *T. circumcincta* and *T. colubriformis* were the dominant worm species.

From 2000 to 2012, individual FEC were recorded between one to three times per production year (considered from September to August), according to the level of infestation found in the periodic monitoring samplings that depended on annual variations in rainfall and temperature. Thus, since the level of infestation was low, no individual measures were carried out between July 2003 and September 2004 and between June 2006 and November 2007. The recording of FEC for the detection of QTL was closed in 2012. In 2015, FEC recording of the new generations of ewes born in the nucleus flock was started again in view of implementing marker-assisted or genomic selection in the Sarda breed. These data were added to the previous set to enhance the power of QTL detection of the analysis presented here.

Finally, 17,594 FEC measurements were recorded on 25 separate dates and on 4477 animals (Table 1). The average number of records per ewe was 3.93 ± 2.2, ranging from 1 (13.4% of animals) to 8 (14.13% of animals); almost half of the ewes (46.7%) had from 3 to 5 records.

**Table 1:** Dates of sampling, number of animals sampled, mean and standard deviation of Fec and LnFec [ln(Fec + 14)]

Date of sampling	Number of animals	Mean Fec EPG	SD (Fec)	Mean lnFec	SD (lnFec)
2000-01-21	939	349.25	563.20	5.11	1.33
2000-06-02	916	110.60	180.43	4.25	1.05
2000-10-02	918	387.23	545.3	5.45	1.09
2001-03-30	890	177.42	199.09	4.77	1.06
2001-09-07	866	301.33	528.23	4.98	1.24
2002-02-02	866	142.41	284.31	4.26	1.21
2003-03-06	699	48.90	118.08	3.54	0.96
2003-07-01	1207	127.93	220.00	4.13	1.28
2004-09-20	748	271.22	308.72	5.15	1.09
2005-07-07	226	177.28	138.97	4.97	0.81
2005-09-05	468	359.55	627.65	4.96	1.42
2006-01-23	857	218.37	355.68	4.68	1.29
2006-07-17	521	356.00	531.96	5.14	1.34
2007-11-13	655	267.16	578.94	4.46	1.51
2008-07-22	645	402.67	797.06	4.95	1.53
2009-06-24	763	848.77	1300.64	5.77	1.57
2010-02-08	554	265.98	446.89	4.83	1.31
2010-05-18	672	161.61	275.56	4.35	1.26
2011-04-06	653	346.36	477.36	5.16	1.29
2012-06-16	609	1590.02	2019.34	6.54	1.56
2015-09-03	528	162.57	246.80	4.45	1.24
2016-07-20	473	506.18	773.90	5.21	1.58
2017-05-20	554	204.57	302.27	4.72	1.18
2018-05-08	674	247.60	465.71	4.29	1.60
2019-02-12	693	389.99	961.97	5.26	1.25

EPG = eggs per g; lnFec = [ln(Fec + 14)]

FEC measurements, that presented a skewed distribution, were log-transformed prior to further analysis using lnFec = ln(FEC + 14).

### Variance components and pseudo-phenotypes for QTL detection

In order to calculate the pseudo-phenotypes for the detection of QTL and to estimate variance components, raw data were analysed by a repeatability model including the permanent environment and additive genetic random effects of individual animals and using the ASReml-R 4.1 software [41]. Environmental fixed effects were the date of sampling, the age of the animal (from 1 to 4 years) and its physiological status at the date of sampling. The levels of the physiological status were built considering the days from parturition and the number of lambs carried or born by the measured ewe in the considered production year. Five classes were considered: ewes without pregnancy and lactation, and thus with no lambs, in the considered production year; ewes sampled within 30 days before or after lambing with one lamb; ewes sampled within 30 days before or after lambing with two or more lambs; lactating ewes with one lamb; and lactating ewes with two or more lambs.

Individual FEC recorded from September to the following dry-off (July) were assigned to the same year of age. Data from animals younger than ten months (570 records), which can be considered without acquired immunisation, were not included so that a measure of the parasite resistance expressed by immunized animals was used. However, 90% of those animals had measurements at older ages which were included in the analysis. Only records from genotyped animals, i.e. born before 2017, were included in the analysis. The final dataset included 16,530 records from 4097 animals recorded on 24 separate dates. Genetic relationships between 4547 animals, including the recorded ewes and their sires and genotyped ancestors, were taken into account by calculating the genomic relationship matrix (GRM) based on 50 k genotypes, following [42] and using the GCTA software [43]. The GRM was then inverted using the Ginv function provided by the Mass R package (version 7.3–51.6), [44] which provides a generalized inverse matrix. Pseudo-phenotypes for QTL detection were then calculated as the average performance deviation (APD) of each ewe as proposed by Usai et al. [27]: i.e. by averaging single animal residuals and summing-up the genetic and permanent environment random predictions.

### QTL detection analysis

The model used for the QTL detection based on 50k SNP data was the same that was applied to the experimental population for milk traits by Usai et al. [27]. It is based on the combined use of LD and linkage analysis (LA) information (LDLA) to estimate the probability of identity-by-descent (IBD) between pairs of gametes of the genotyped individuals at the investigated position. First, the paternal and maternal inherited gametes of the genotyped individuals were reconstructed by the LD multilocus iterative peeling method [27, 45] by exploiting the genotypes and the familial structure of the population. Then, the base gametes of the population were identified as the gametes inherited from an ungenotyped parent and corresponded to: the gametes of the 10 F1 rams and of the 74 Sarda (grand) sires, the maternal or paternal gametes of the 43 ewes with an unknown sire or dam, respectively, and the maternal gametes of the 928 back-cross ewes and of the 108 Sarda (grand) sires for which only the sire was genotyped. The 1247 base haplotypes (BH) were further divided according to their breed of origin in BH^L^ (the 10 Lacaune paternal gametes carried by the F1 rams) and BH^S^ (the remaining 1237 Sarda gametes). Finally, the remaining parental gametes of the genotyped animals which carried, at each position, an allele inherited from one out of the 1247 original BH were considered as replicates of BH (RH).

The IBD between pairs of BH were estimated by LD analysis ($IBD_{LD}$) based on the extent of identity-by-state (IBS) around the investigated position [46]. The $IBD_{LD}$ between BH^S^ and BH^L^ were assumed to be null. The IBD between BH and RH were estimated by LA analysis ($IBD_{LA}$) given the known gametic phases and the pedigree information [27, 46–48]. The IBD between pairs of RH were, thus, calculated as the combination of $IBD_{LD}$ and $IBD_{LA}$ ($IBD_{LDLA}$). This allowed the construction, at each 50k SNP position l, of a matrix (${\mathbf{G}}_{l}^{IBD}$) allocating IBD between RH carried by phenotyped ewes. Moreover, in order to account for the polygenic effects, a matrix of genome-wide IBD between gametes (${\mathbf{G}}_{g}^{IBD}$) was constructed by averaging elements of ${\mathbf{G}}_{l}^{IBD}$ across all the investigated SNP positions. At this stage, Usai et al. [27] proposed the use of principal component analysis (PCA) to summarize the information of ${\mathbf{G}}_{l}^{IBD}$ and ${\mathbf{G}}_{g}^{IBD}$. The aim of using PCA was to overcome issues related to the non-positive definite status of ${\mathbf{G}}_{l}^{IBD}$ and to limit the computational needs in handling both the IBD matrices. In fact, PCA led to a dramatic reduction in the number of effects to be estimated, so that the principal components from ${\mathbf{G}}_{l}^{IBD}$ and ${\mathbf{G}}_{g}^{IBD}$ can be included in the model as fixed effects. The final model does not include random effects other than the residuals and is solved by a weighted least square method.

At each 50k SNP position l the model is the following:

1 $$ {\mathbf{y}} = \bf{1}{\upmu } + {\mathbf{ZV}}_{{\mathbf{l}}} {{\boldsymbol{\upbeta}}}_{{\mathbf{l}}} + {\mathbf{ZV}}_{{\mathbf{g}}} {{\boldsymbol{\upalpha}}}_{{\mathbf{l}}} + {{\boldsymbol{\upvarepsilon}}}, $$

where ${\mathbf{y}}$ is a vector of APD of ${\text{n}}_{{\text{p}}}$ phenotyped ewes for LnFec; $\mu$ is the overall mean; ${{\varvec{\upbeta}}}_{l}$ is a vector of the fixed effects of the ${\text{n}}_{{{\text{PC}}_{l} }}$ principal components that explain more than 99% of the within-breed variation (${\text{PC}}_{l}$) of the IBD probability matrix ${\mathbf{G}}_{l}^{IBD}$, i.e. ${{\varvec{\upbeta}}}_{l}$ summarizes the effects of the gamete at the QTL position $l$; ${{\varvec{\upalpha}}}_{l}$ is a vector of the fixed effects of the ${\text{n}}_{{{\text{PC}}_{g} }}$ principal components that explain more than 99% of the variation (${\text{PC}}_{g}$) of the genome-wide IBD probability matrix ${\mathbf{G}}_{g}^{IBD}$, i.e. ${{\varvec{\upalpha}}}_{l}$ summarizes the polygenic effects of the gametes; $\bf{1}$ is a vector of ${\text{n}}_{{\text{p}}}$ ones; ${\mathbf{Z}}$ is a ${\text{n}}_{{\text{p}}} \times {\text{n}}_{{{\text{RH}}}}$ incidence matrix relating phenotypes with RH; ${\mathbf{V}}_{l}$ is a ${\text{n}}_{{{\text{RH}}}} \times {\text{n}}_{{{\text{PC}}_{l} }}$ matrix including the ${\text{PC}}_{l}$ scores of RH that summarize the IBD probabilities between the gametes at the considered position; ${\mathbf{V}}_{g}$ is a ${\text{n}}_{{{\text{RH}}}} \times {\text{n}}_{{{\text{PC}}_{g} }}$ matrix including the ${\text{PC}}_{g}$ scores of RH; ${{\varvec{\upvarepsilon}}}$ is a vector of ${\text{n}}_{{\text{p}}}$ residuals assuming that ${{\varvec{\upvarepsilon}}}\sim {\text{N}}\left( {\mathbf{0},\sigma_{{\upvarepsilon }}^{2} {\mathbf{R}}^{ - 1} } \right)$ with ${\mathbf{R}}$ being a diagonal matrix with the APD’s reliability ($r$) as diagonal element. Reliabilities were calculated as $r_{{\text{i}}} = 1 - {\text{se}}\left( {{\hat{\text{a}}}_{{\text{i}}} } \right)^{2} /\sigma_{{\text{a}}}^{2}$, from a repeatability linear model ${\text{y}}_{{{\text{ij}}}} = {\text{a}}_{{\text{i}}} + {\text{e}}_{{{\text{ij}}}}$, where ${\text{y}}_{{{\text{ij}}}}$ is the performance deviation ${\text{j}}$ adjusted for the fixed effects estimated with the full animal model of ewe ${\text{i}}$, ${\text{a}}_{{\text{i}}}$ is the random ewe effect assuming that ${\mathbf{a}}\sim {\text{N}}\left( {\mathbf{0}, \sigma_{{\text{a}}}^{2} {\mathbf{I}}} \right)$, and ${\text{e}}_{{{\text{ij}}}}$ is the corresponding error, assuming that ${\mathbf{e}}\sim {\text{N}}\left( {\mathbf{0}, \sigma_{{\text{e}}}^{2} {\mathbf{I}}} \right)$. Details on how the PC scores of the ${\mathbf{V}}_{l}$ and ${\mathbf{V}}_{g}$ matrices were calculated are in [27].

Since the IBD between segments of different breed origin (i.e. replicates of ${\text{BH}}^{{\text{S}}}$ and ${\text{BH}}^{{\text{L}}}$) was set to 0, the PCA generated two sets of breed-specific ${\text{PC}}_{l}$. Thus, the matrix ${\mathbf{V}}_{l}$ can be written as $\left[ {{\mathbf{V}}_{l}^{{\text{S}}} {\mathbf{V}}_{l}^{{\text{L}}} } \right]$ and the vector ${{\varvec{\upbeta}}}_{l}^{^{\prime}}$ as $\left[ {{{\varvec{\upbeta}}}_{l}^{{^{\prime}{\text{S}}}} {{\varvec{\upbeta}}}_{l}^{{^{\prime}{\text{L}}}} } \right]$, where ${\mathbf{V}}_{l}^{{\text{S}}}$ and ${\mathbf{V}}_{l}^{{\text{L}}}$ are the ${\text{PC}}_{l}$ summarising IBD probabilities between the gametes of Sarda and Lacaune origin and ${{\varvec{\upbeta}}}_{l}^{{\text{S}}}$ and ${{\varvec{\upbeta}}}_{l}^{{\text{L}}}$ the corresponding effects.

The final aim of this work was to identify QTL segregating in the Sarda breed and to search for candidate genes and causative mutations by functional annotation and association analysis of the imputed Sarda sequence data in the identified regions. Thus, at each SNP position, we tested the null hypothesis that the effects of the principal components that explain 99% of the variability due to the Sarda gametes are zero ($H_{0}$: ${{\varvec{\upbeta}}}_{{\mathbf{l}}}^{{\mathbf{S}}}$ = 0) by an F-test that compares the sums of squared residuals of the full model in Eq. (1) and of the following reduced model including all the other effects:

$$ {\mathbf{y}} = \bf{1}{\upmu } + {\mathbf{ZV}}_{{\mathbf{l}}}^{{\mathbf{L}}} {{\boldsymbol{\upbeta}}}_{{\mathbf{l}}}^{{\mathbf{L}}} + {\mathbf{ZV}}_{{\mathbf{g}}} {{\boldsymbol{\upalpha}}}_{{\mathbf{l}}} + {{\boldsymbol{\upvarepsilon}}}^{*} . $$

The Bonferroni correction for multiple testing was used to estimate the threshold corresponding to the genome-wise (GW) significance level. To be conservative, we omitted the LD between SNPs, and calculated the nominal P-value for each tested position as $P_{nominal} = \frac{{P_{GW} }}{n Test}$, were $P_{GW}$ is the genome-wise significance level chosen for the analysis (0.05) and $n Test$ is the number of tested positions (43,390). The negative logarithm of $P_{nominal}$ resulted in a threshold of $- {\text{log}}_{10} \left( {Pvalue} \right)$ equal to 5.938, which was rounded to 6.

Significant positions identified on the same chromosome were clustered into QTL regions (QTLR) in order to account for linkage between SNPs. As proposed by Usai et al. [27], the correlations between $\widehat{{{\varvec{y}}_{{{\varvec{Q}}_{{\varvec{l}}} }} }} = {\mathbf{ZV}}_{l} {\widehat{\varvec{\upbeta }}}_{l}$ (corresponding to the portion of phenotypes predicted in the model by the QTL effect) were calculated for all pairs of significant SNPs on the same chromosome. The most significant SNP on the chromosome was taken as the peak of the first QTLR. Peaks that identified a further QTLR on the same chromosome were iteratively detected as the significant SNPs showing correlations lower than 0.15 with the already defined QTLR peaks. The remaining significant positions were assigned to the QTLR with which they had the highest correlation. Moreover, with the aim of appreciating the relative potential impact of a marker-assisted selection approach, we calculated the ANOVA estimator of the QTL variance for the most significant position of each QTLR as:

$$ \widehat{{\sigma_{qtlS}^{2} }} = \frac{{\frac{{SSE_{R} - SSE_{F} }}{{nPC_{S} }} - \frac{{SSE_{F} }}{{np - nPC_{g} - nPC_{L} - nPC_{S} - 1}}}}{{\frac{np}{{nPC_{S} }}}}, $$

where $$ SSE_{F} = \left\lceil{\mathbf{y}} - \left( {\bf{1}{{\boldsymbol{\upmu}}} + {\mathbf{ZV}}_{{\mathbf{l}}}^{{\mathbf{L}}} {{\boldsymbol{\upbeta}}}_{{\mathbf{l}}}^{{\mathbf{L}}} + {\mathbf{ZV}}_{{\mathbf{l}}}^{{\mathbf{S}}} {{\boldsymbol{\upbeta}}}_{{\mathbf{l}}}^{{\mathbf{S}}} + {\mathbf{ZV}}_{{\mathbf{g}}} {{\boldsymbol{\upalpha}}}_{{\mathbf{l}}} } \right)
\right\rceil^{^{\prime}}  \left\lceil{\mathbf{y}} - \left( {\bf{1}{{\boldsymbol{\upmu}}} + {\mathbf{ZV}}_{{\mathbf{l}}}^{{\mathbf{L}}} {{\boldsymbol{\upbeta}}}_{{\mathbf{l}}}^{{\mathbf{L}}} + {\mathbf{ZV}}_{{\mathbf{l}}}^{{\mathbf{S}}} {{\boldsymbol{\upbeta}}}_{{\mathbf{l}}}^{{\mathbf{S}}} + {\mathbf{ZV}}_{{\mathbf{g}}} {{\boldsymbol{\upalpha}}}_{{\mathbf{l}}} } \right) \right\rceil$$ is the sum of squared residuals of the full model including the Sarda PC at the peak position; and $$SSE_{R} = \left\lceil{\mathbf{y}} - \left( {\bf{1}{{\boldsymbol{\upmu}}} + {\mathbf{ZV}}_{{\mathbf{l}}}^{{\mathbf{L}}} {{\boldsymbol{\upbeta}}}_{{\mathbf{l}}}^{{\mathbf{L}}} + {\mathbf{ZV}}_{{\mathbf{g}}} {{\boldsymbol{\upalpha}}}_{{\mathbf{l}}} } \right)\right\rceil^{^{\prime}} \left\lceil{\mathbf{y}} - \left( {\bf{1}{{\boldsymbol{\upmu}}} + {\mathbf{ZV}}_{{\mathbf{l}}}^{{\mathbf{L}}} {{\boldsymbol{\upbeta}}}_{{\mathbf{l}}}^{{\mathbf{L}}} + {\mathbf{ZV}}_{{\mathbf{g}}} {{\boldsymbol{\upalpha}}}_{{\mathbf{l}}} } \right)\right\rceil$$ is the sum of squared residuals of the reduced (without the Sarda PC) model; and $$nPC_{S}$$ and $$nPC_{L}$$ are the number of PC summarising the IBD probabilities between the gametes of Sarda and Lacaune origin, respectively; and $$nPC_{g}$$ is the number of PC extracted from the genome-wide IBD probability matrix.

The ratio between the ANOVA estimators of the QTL variances ($\widehat{{\sigma_{qtlS}^{2} }}$) and the total phenotypic variance of the pseudo-phenotypes was calculated for the peak of each QTLR.

### Analysis of sequence data

The QTLR as defined above or the 2-Mb intervals that surround the most significant locations when only one 50k SNP was significant, were further investigated using information from whole-genome sequence (WGS) data. Biallelic SNPs falling in these target QTLR were extracted from the assembled sequences of the re-sequenced animals as vcf-files. First, a functional annotation of the SNPs identified by WGS was performed using the NCBI 4.0 sheep genome annotation release 102 and the snpEff software v4.3.t [49]. Then, the parental gametes of the phenotyped ewes were imputed from 50 k data to WGS. The first step of the imputation procedure was to reconstruct the phase of each gamete $i$ carried by the sequenced animals ($h_{i}^{Q}$) that consisted in estimating the probability of carrying the reference $P\left( {h_{il}^{Q} = R} \right)$ and the alternative $P\left( {h_{il}^{Q} = A} \right)$ allele at each WGS SNP position l based on the genotype information from sequencing and the IBD between gametes at the neighbouring SNP 50k. Then, at each WGS SNP position l of the parental gamete $j$ carried by each of the none-sequenced phenotyped ewes, we inferred the probabilities of carrying the reference $P\left( {h_{jl}^{p} = R} \right)$ and the alternative allele $P\left( {h_{jl}^{p} = A} \right)$ based on the gametic phase of sequenced animals and the IBD between gametes of sequenced animals with gametes of the phenotyped ewes [50]. The accuracy of the imputation was calculated as the correlation between the probability for an imputed WGS SNP allele at each 50k SNP position and the actual occurrence of the same allele given the 50k genotyping information and the gametic phase reconstructed in the previous analysis. Moreover, to verify that the imputed data could be used for the association analysis, the information content of each WGS SNP for all imputed gametes was calculated as the squared difference of the allele probabilities $\left[ {P\left( {h_{jl}^{p} = R} \right) - P\left( {h_{jl}^{p} = A} \right)} \right]^{2}$. These statistics were averaged across positions and gametes.

Finally, an association analysis was run in the target regions, by regressing the pseudo-phenotypes on the allele dosage calculated as the sum of the probabilities of carrying the reference allele in the paternal and maternal gametes predicted by imputation. The allele dosage was used instead of the genotype probabilities since it allows the direct estimation of the additive substitution effect of the reference allele with just one regressor in the model. However, the genotype probabilities imply a multiple regression model and are more adapted for estimating non-additive effects. As in Eq. (1), the model included the PC extracted from the genome-wide IBD probability matrix to adjust for the polygenic background.

An F test was performed to calculate the P-values of each tested WGS SNP. The analysis was performed in order to identify the most relevant WGS SNPs, which were selected by setting the threshold of $- {\text{log}}_{10} \left( {Pvalue} \right)$ equal to the maximum per region minus 2.

### Searching for candidate genes

Genes that harboured variants with a potential functional impact or variants that showed the highest P-values identified in the previous analyses, were compared with functional candidate genes selected from QTL or gene expression studies related to GIN resistance. In particular, we took advantage of the recent summary provided by [51] in which a deep review of the latest literature on the subject was performed. They identified 11 SNP chip-based QTL detection analyses (based on GWAS, LA, LDLA, selection sweep mapping or regional heritability mapping methods) from which they extracted 230 significantly associated genomic regions. Moreover, they proposed a list of 1892 genes reported as highly expressed or differentially expressed after GIN infection in sheep by 12 different experiments in the field. QTL regions and GIN activated genes proposed by [51] were remapped from the *Ovis aries* genome 3.1 assembly to the Oar4.0 version by using Biomart and NCBI remapping services for comparison with our results.

Finally, we performed an over-representation analysis (ORA) of gene ontology (GO) biological process terms of the genes harboring significant mutations or mutations with functional consequences on the transcripts. We performed the ORA with the web-based software WebGestalt [52]. Gene symbols of human gene orthologues were retrieved from the OrthoDB v10 data base [53] starting from the NBCI ID of sheep genes from the *Ovis aries* annotation release 102. The human genome protein coding database was taken as reference and the following parameters were used for the analysis: default statistical method (hypergeometric); minimum number of genes included in the term = 5, multiple test adjustment = BH method (Benjamini–Hochberg FDR). The ten top categories were retained based on FDR rank.

## Results

### Variance components

Table 2 shows the variance component estimates obtained with the repeatability animal model. The heritability and repeatability estimates of lnFec were 0.21 ± 0.015 and 0.27 ± 0.012, respectively (Table 2).

**Table 2:** Estimates and standard errors of genetic, permanent environment (Pe) and residual variances and repeatability (Rp) and heritability (h^2^) estimates for LnFec

	Component	Standard error
Genetic (ln^2^)	0.341	0.026
Pe (ln^2^)	0.097	0.016
Residual (ln^2^)	1.187	0.015
Rp	0.270	0.012
h^2^	0.210	0.015

### QTL detection analysis

**Fig. 1:**
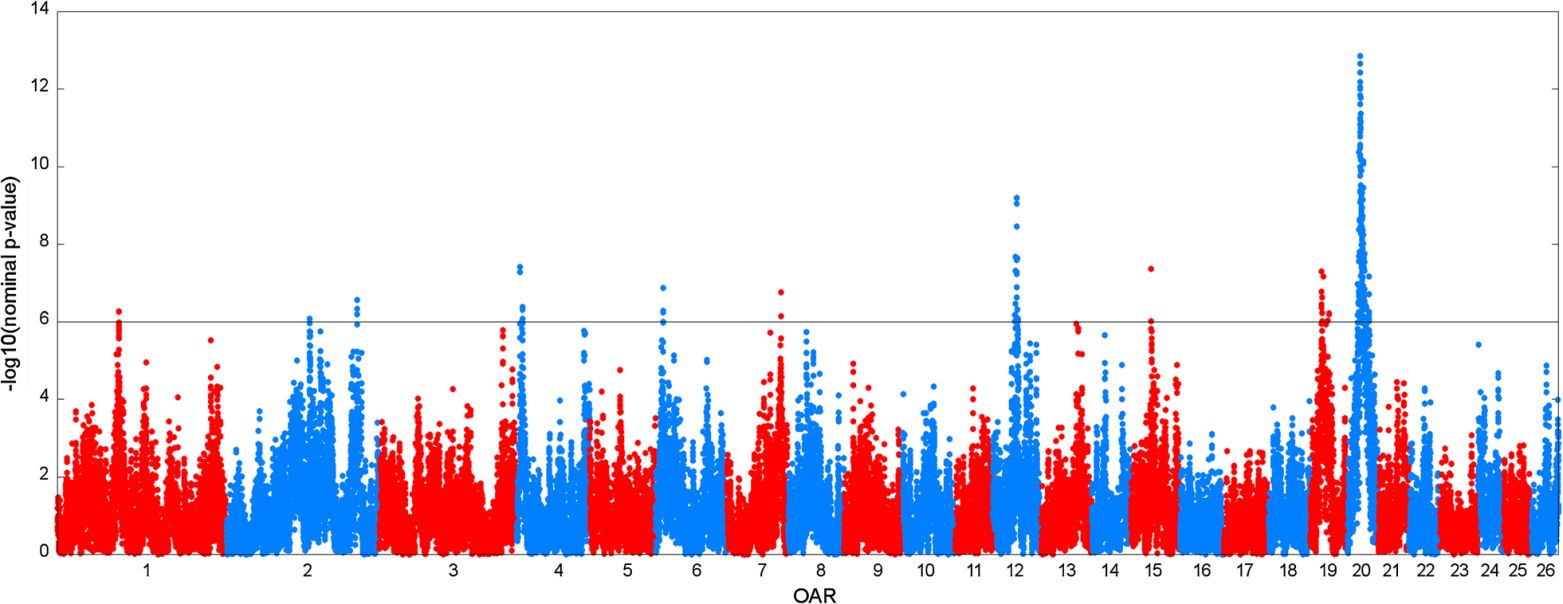
Manhattan plot of the $- {\text{log}}_{10} \left( {Pvalue} \right)$ corresponding to the null hypothesis that the effects of principal components that explain 99% of the variability due to the Sarda base gametes at each locus are zero. The grey line indicates the 0.05 genome-wide significance threshold determined by Bonferroni correction for 43,390 tests

Figure 1 presents the Manhattan plot of the $- {\text{log}}_{10} \left( {Pvalue} \right)$ corresponding to the null hypothesis that the effects of PC that explain 99% of the variability due to the Sarda base gametes at each locus (43,390 SNPs) are zero. Two hundred and two SNPs encompassed the 5% genome-wide significant threshold. With the exception of *Ovis aries* chromosome (OAR) 1, on which only one significant location was found, many significant SNPs mapped to the same chromosome. After clustering the significant locations on the same chromosome, ten QTLR were defined on nine chromosomes (Table 3). The ratio between the ANOVA estimator of the QTL variance and the total phenotypic variance ranged from 0.0087 to 0.0176.

**Table 3:** QTL regions from the LDLA analysis

QTLR	OAR	Range (bp)	nSNP	Pos (bp)	$$- \log_{10} \left( {{Pvalue}} \right)$$	$${\raise0.7ex\hbox{${\widehat{{{\boldsymbol{\sigma}}_{{{\varvec{qtlS}}}}^{2} }}}$} \!\mathord{\left/ {\vphantom {{\widehat{{{\boldsymbol{\sigma}}_{{{\varvec{qtlS}}}}^{2} }}} {{\boldsymbol{\sigma}}_{{{\varvec{APD}}}}^{2} }}}\right.\kern-\nulldelimiterspace} \!\lower0.7ex\hbox{${{\boldsymbol{\sigma}}_{{{\varvec{APD}}}}^{2} }$}}$$
Q_01_1	1	99,988,914–99,988,914	1	99,988,914	6.256	0.0087
Q_02_1	2	135,598,790–135,598,790	1	135,598,790	6.078	0.0093
Q_02_2	2	213,089,849–213,166,468	3	213,135,270	6.554	0.0093
Q_04_1	4	5,252,005–9,837,812	6	5,386,849	7.404	0.0111
Q_06_1	6	12,638,149–12,695,314	3	12,695,314	6.864	0.0099
Q_07_1	7	88,040,710–88,085,726	2	88,040,710	6.762	0.0098
Q_12_1	12	35,973,543–41,153,795	19	39,430,517	9.191	0.0125
Q_15_1	15	34,024,406–34,081,200	2	34024406	7.362	0.0110
Q_19_1	19	18,933,658–31,429,916	11	18,983,777	7.302	0.0104
Q_20_1	20	16,796,770–36,098,292	154	21,170,858	12.861	0.0176

QTLR: QTL region identifier; OAR *Ovis aries* autosome; Range: position, in bp from the ovine genome assembly v4.0, of the first and last significant SNP of the QTL region; nSNP number of SNPs from the OvineSNP50 Beadchip exceeding the 0.05 genome-wide significance threshold $$- {\text{log}}_{10} \left( {Pvalue} \right) > 6$$; Pos: position of the most significant SNP in bp; $$- {\text{log}}_{10} \left( {Pvalue} \right)$$: negative logarithm of the p-value of the most significant SNP; $${\raise0.7ex\hbox{${\widehat{{\sigma_{qtlS}^{2} }}}$} \!\mathord{\left/ {\vphantom {{\widehat{{\sigma_{qtlS}^{2} }}} {\widehat{{\sigma_{APD}^{2} }}}}}\right.\kern-\nulldelimiterspace} \!\lower0.7ex\hbox{${\widehat{{\sigma_{APD}^{2} }}}$}}$$: ratio between the ANOVA estimator of the QTL variance at peak position ($$\widehat{{\sigma_{qtlS}^{2} }}$$) and the total phenotypic variance of the pseudo-phenotypes ($$\widehat{{\sigma_{APD}^{2} }}$$)

The most significant location ($- {\text{log}}_{10} \left( {Pvalue} \right)$ = 12.861) was in a large region on OAR20, that covered almost 20 Mb and included 154 significant SNPs. Correlations between $\widehat{{{\varvec{y}}_{{{\varvec{Q}}_{{\varvec{l}}} }} }}$ at the peak position and the other 153 significant locations were always higher than 0.25. The second most significant peak was on OAR12 in a QTLR spanning 5.18 Mb and including another 18 significant SNPs, with correlations between $\widehat{{{\varvec{y}}_{{{\varvec{Q}}_{{\varvec{l}}} }} }}$ greater than 0.46. The third QTLR in order of significance was at the beginning of OAR4, spanned 4.6 Mb and included six SNPs. Eleven SNPs on OAR19 exceeded the 5% genome-wide significance threshold. Although the two most distant SNPs defined an interval of about 12.5 Mb, all the SNPs clustered in the same QTLR, since the correlations between $\widehat{{{\varvec{y}}_{{{\varvec{Q}}_{{\varvec{l}}} }} }}$ were always higher than 0.48. Other QTLR (approximately 500 to 700 kb long and including from 1 to 3 significant SNPs) were identified on OAR15, 6, 7 and 2. An additional significant SNP, ~ 100 Mb apart from the previous one, was also identified on OAR2. The last QTLR was defined in the 2-Mb interval surrounding the single significant SNP on OAR1.

### Analysis of sequence data

QTLR, rounded to the closest Mb, were further investigated with WGS data. Overall, 712,987 biallelic SNPs were extracted from the target regions. Among these, 649,054 were already known in the European Variation Archive (EVA, ftp://ftp.ebi.ac.uk/pub/databases/eva/rs_releases/release_1/by_species/Sheep_9940/GCA_000298735.2), while 63,933 (8.96%) were novel variants, without an associated rs identifier.

The average mutation rate ranged from 7711 to 14,428 SNPs per Mb. Accuracy of imputation at the 50 k SNP positions ranged from 0.990 on OAR6 to 0.979 on OAR7 (Table 4). The imputation process resulted in an average information content across gametes and QTLR of 0.976 ± 0.17, which ranged from 0.967 ± 0.02 for OAR4 to 0.985 ± 0.14 for OAR12. Based on such informativeness, we performed an association analysis at each polymorphic site from WGS (Table 5). Graphical comparison between Manhattan-plots of LDLA and WGS-based data association analysis are reported in Additional file 1: Figs. S1–S10.

**Table 4:** Description of the QTL regions from whole-genome sequences and results of the imputation procedure

QTLR	Interval			IC	Accuracy	
	Range (Mb)	nSNPs^a^	MR (nSNPs/Mb)	Mean ± SD	nSNPs^b^	Mean ± SD
Q_01_1	99–100	15,410	7711	0.983 ± 0.017	33	0.983 ± 0.026
Q_02_1	135–137	21,708	10,859	0.973 ± 0.015	39	0.986 ± 0.009
Q_02_2	212–214	24,361	12,181	0.982 ± 0.013	35	0.995 ± 0.004
Q_04_1	4–10	86,568	14,428	0.967 ± 0.020	113	0.987 ± 0.011
Q_06_1	12–14	26,466	13,234	0.973 ± 0.014	40	0.990 ± 0.005
Q_07_1	87–89	26,375	13,188	0.974 ± 0.016	43	0.979 ± 0.034
Q_12_1	35–42	54,785	7827	0.985 ± 0.014	119	0.995 ± 0.006
Q_15_1	33–35	21,528	10,764	0.972 ± 0.018	34	0.986 ± 0.009
Q_19_1	18–32	167,487	11,963	0.972 ± 0.019	222	0.987 ± 0.017
Q_20_1	16–37	268,299	12,778	0.976 ± 0.021	365	0.987 ± 0.028
Total	–	712,987	11,884	0.976 ± 0.017	1043	0.988 ± 0.021

QTLR: QTL region identifier; Range: start and end position of the explored interval in Mb from the ovine genome assembly v4.0; nSNP^a^: number of polymorphic sites from sequence data; MR: mutation rate, i.e. number of SNPs per Mb; nSNP^b^: number of polymorphic sites from both the OvineSNP50 Beadchip and sequence data used for calculating the accuracy of imputation; IC: information content and Accuracy: accuracy of imputation of gametes carried by phenotyped ewes

**Table 5:** Results of the association analysis based on imputed alleles at the polymorphic sites from WGS

QTLR	OAR	SNP ID	Pos	$- \log_{10} \left( {{Pvalue}} \right)$
Q_01_1	1	rs430083769	100,326,208	6.4402
Q_02_1	2	rs416887054	135,331,051	6.6044
Q_02_2	2	rs418850058	213,396,760	5.5896
Q_04_1	4	rs430604472	8,686,421	8.2004
Q_06_1	6	rs400533049	13,159,311	6.9217
Q_07_1	7	rs398163681	87,832,764	6.6496
Q_12_1	12	rs405838891	41,043,088	8.0020
Q_15_1	15	rs427631513	34,330,615	4.9680
Q_19_1	19	rs420265308	19,228,546	8.8439
Q_20_1	20	rs404860665	26,158,816	13.2036

QTLR: QTL region identifier; OAR *Ovis aries* autosome; SNP ID: SNP identifier; Pos: position of the most significant SNP in bp based on the ovine genome assembly v4.0.; -log10 (Pvalue) negative logarithm of the P-value of the most significant SNP

QTL on OAR4, 12, 19 and 20 remained the most significant. As in the LDLA analysis, the test statistic profile in the WGS analysis was not unimodal and, in some cases, the most significant positions were at different locations compared to the previous analysis. Thus, on OAR4 the peak from the WGS association analysis mapped at 8,686,421 bp, closer to the second peak and almost 3.3 Mb from the most significant position identified with LDLA. Similarly, on OAR12, the WGS peak position was at 41,043,088 bp, 1.6 Mb from the LDLA peak and close to a SNP from the OvineSNP50 Beadchip which did not reach genome-wide significance with LDLA ($- {\text{log}}_{10} \left( {Pvalue} \right)$ = 5.79). On OAR19, the most significant position in the LDLA and WGS analyses were only 467 kb apart, although the explored region was 14 Mb long and showed several peaks in both analyses. As far as the QTLR on OAR20 was concerned, the most significant position in the WGS association analysis, was almost 5 Mb distant from the LDLA peak. However, the other WGS significant SNPs were close to the LDLA peak. Indeed, the second peak from WGS was only 68 kb apart from the LDLA peak. Moreover, the SNPs from the OvineSNP50 Beadchip which were closer to the second (rs416381272) and third (rs411905117) significant WGS peaks also ranked third and second in the LDLA analysis. In the other analysed QTLR, with a lower significance level and smaller number of significant SNPs, peak positions from WGS data were within a distance of 500 kb from the LDLA peaks. Finally, while nominal P-values remained similar in the two analyses for most of the investigated regions, an evident drop of significance was observed on OAR15, where the $- {\text{log}}_{10} \left( {Pvalue} \right)$ dropped from 7.36, in the LDLA analysis to 4.97 in the WGS based association analysis.

As far as the functional annotation was concerned, SNPeff provided 2,250,514 effects for the 712,987 analysed SNPs in the explored 60 Mb, since a variant can affect two genes and a gene can have multiple transcripts (Table 6).

**Table 6:** Summary of the genomic features in the investigated regions

QTLR	tRNA	lncRNA	miRNA	misc_RNA	Pseudogene	Protein coding (n transcr)
Q_01_1	1	10		1	12	65 (153)
Q_02_1	1	2			1	11 (38)
Q_02_2	1	1				3 (16)
Q_04_1	1	6			2	30 (74)
Q_06_1	1	5			1	10 (83)
Q_07_1	2	6				3 (22)
Q_12_1	3	12	1		7	77 (170)
Q_15_1		3			2	23 (43)
Q_19_1	1	12		1	5	27 (118)
Q_20_1	43	46		1	47	423 (761)
Total	54	103	1	3	77	672 (1478)

QTLR: QTL region identifier; lncRNA: long non coding RNAs; miRNA; microRNAs; misc_RNA: miscellaneous RNA; pseudogene: genes with pseudogene biotype; protein coding: number of genes (and number of transcripts) that contains an open reading frame (ORF)

The number of effects by impact (high, moderate, modifier and low), type and region according to SNPeff classification is reported in Additional file 2: Tables S1–S10. Among the SNPs that affect transcripts, 0.8 to 1% of them per region, concerned pseudogenes and were not considered. In addition, variants that were in intergenic regions (from 4.2 to 27.4% of the predicted effects per QTLR) were not further investigated.

Finally, we focused on variants that were classified as having a high impact on the transcript of protein coding genes (classified by SNPeff as: splice_acceptor_variant; splice_donor_variant; start_lost; stop_gained; stop_lost) or a moderate impact (which were all predicted as having a missense effect in our case, i.e. variants that change one or more bases, resulting in a different amino acid sequence but the length of which is preserved). On the whole, 3538 polymorphisms were predicted to cause high-impact or missense effects (340 and 9105 effects, respectively) on the multiple transcripts of 530 protein coding genes. A detailed description of the classification of the retained variants is in Additional file 3: Table S11.

The ten most significant SNPs from the WGS analysis were all classified as modifier, since they were either intergenic or intronic (see Additional file 4: Table S12), and thus had no effect on the transcript. None of the high-impact variants showed high significance levels. Indeed, only four missense variants encompassed the empirical threshold of $- {\text{log}}_{10} \left( {Pvalue} \right)$ equal to the maximum per region minus 2: one affected three transcripts of the *CIART* (*circadian associated repressor of transcription*) gene on OAR1 (rs159646335) and three affected the transcript of the *OTOG* (*otogelin*) gene on OAR15 (rs420057627, rs401738285 and rs422155776).

The 530 genes that harbored high or moderate (missense effect) impact variants and another 13 genes with polymorphisms encompassing the empirical threshold of $max\left( { - {\text{log}}_{10} \left( {Pvalue} \right)} \right) - 2$ were submitted to an enrichment analysis of GO biological process terms. Of the 543 genes considered, 50 did not have a human ortholog in the OrthoDb database [53] and 493 mapped to 442 human genes, since 53 shared the same human ortholog. Finally, 376 genes were annotated to the selected functional categories (GO biological process) and were used for the enrichment analysis.

None of the GO terms identified by the enrichment analysis from the biological process database was significantly enriched. The ten most abundant terms (see Additional file 5: Table S13) identified (interferon-gamma-mediated signaling pathway; sialic acid transport; T cell receptor signaling pathway; activation of immune response; positive regulation of immune system process; regulation of immune system process; immune response-activating cell surface receptor signaling pathway; immune response-regulating signaling pathway; innate immune response; and defense response) were further clustered into three superior categories according to the Weighted set cover method for redundancy reduction available in Genstalt [52]: sialic acid transport; regulation of immune system process; and defense response. The last two categories, which clearly relate to resistance to diseases, included 53 and 56 genes, respectively, 36 of which enriched both terms. Among the genes in one of these two GO higher categories, 12 were also in the list of GIN activated genes provided by Chitneedi et al. 2020 [51]: *CTSS* on OAR1, *TNFRSF1B* and *SELE* on OAR12, *IL5RA* on OAR19, *IL17A*, *IL17F*, *TRIM26*, *TRIM38*, *TNFRSF21*, *LOC101118999*, *VEGFA*, and *TNF* on OAR20.

## Discussion

The heritability estimate of lnFec in this study was low to moderate and consistent with previous studies in adult ewes, which reported heritabilities of FEC, after appropriate logarithmic or squared root transformation, ranging from 0.09 [54] to 0.21 [12] and 0.35 [14]. On the contrary, the repeatability estimate was higher with the permanent environmental variance equal to 6% of the total phenotypic variance. Aguerre et al. [14] did not find significant differences between heritability and repeatability estimates in naturally-infected ewes and suggested that individual variability was mainly due to differences in the genetic background rather than in differences in the immune history of the animals. Although the characterisation of worm species in individual samples was not systematically performed in our experiment, it has been demonstrated that resistance to different species of nematodes tend to be interrelated, with genetic correlations between FEC values from different species or genera of parasites being generally close to 0.5 or higher in some cases [55, 56]. Moreover, it has been shown that sheep that are selected on the basis of their response to artificial challenges respond similarly when exposed to natural infection, and a high positive genetic correlation was estimated between FEC recorded under artificial or natural infection [14, 57]. Such evidence and the heritability estimate found in our study suggest that genetic selection for parasitism resistance could be considered in the Sarda breed.

The LDLA analysis identified 202 genomic positions that were significantly associated to FEC. We grouped these positions into regions based on the correlations between the predicted effects of the QTL. Five of the ten identified QTLR (OAR4, 7, 12, 19, 20) overlapped with regions that were shown to be associated to traits related to GIN resistance in previous SNPs based studies. In particular, the QTLR on OAR4, 12, 19 and 20 overlap with significant windows identified by [21] in a meta-analysis based on the regional heritability mapping method on data including the first two generations of our experimental population. QTLR on OAR19 has also been found to be significantly associated to FEC measured in lambs [58], while several positions on OAR20 have been indicated as associated to susceptibility to parasites in other studies [17, 19, 20]. The QTLR on OAR7 falls in a region that was identified in a breed of sheep adapted to tropical climate [59] and is close to a signature of selection detected by comparing two breeds selectively bred for high and low FEC [22]. The regions associated to resistance to nematode infection on OAR2 [20, 58, 59], OAR6 [20, 23, 59, 61] and OAR15 [58, 61] were found in several studies but only our first QTLR on OAR2 (Q_02_1), was close to previously reported significant positions [20, 58, 59].

QTL associated to nematode resistance have been identified on almost all the ovine chromosomes (see [10, 62] and [51] for a recent summary) for a recent summary). However, the comparison of results between studies is complex due to the variability of the breeds and nematode species analyzed, and to the use of different statistical approaches. It is likely that resistance to GIN is a complex trait that is determined by a large number of genes [63], and, to date, no major gene has been identified.

In this study, we examined whether combining the significant results obtained from an association analysis of accurate imputed data with the functional annotation of SNPs within target regions was advantageous. The original idea was to verify if considering the significance levels of SNPs was useful to pinpoint functional variants with a potential impact on candidate genes that are identified based on their ontological classification or that are differentially expressed in studies that analyze susceptibility differences of sheep to nematodes. All these results are summarized in Additional file 3: Table S11.

The WGS association analysis was not able to provide a definite significance profile within QTLR. In all the QTLR, the number of peaks still remained large, and often the distance between them was quite big. This is likely a consequence of the large size of the chromosomal segments with high correlations between $\widehat{{{\varvec{y}}_{{{\varvec{Q}}_{{\varvec{l}}} }} }}$ that reveals high LD levels within QTLR. Moreover, none of the most significant SNPs showed a functional effect on the genes’ transcript. This result can be in part due to the fact that we focused on intragenic regions of protein coding genes, whereas it has been suggested that a large part of the genetic variability of quantitative traits lies in regulatory regions or in non-protein coding regions, which are, however, very poorly annotated in the ovine genome.

However, our results indicate that the QTLR located on OAR12, 19 and 20 are strongly involved in the complex mechanism of resistance of sheep to GIN. Not only these regions harbor the most significant SNPs in both the LDLA and WGS analyses, but they have also been reported in the literature either from other QTL detection analyses and from studies on GIN resistance based on differential gene expression. In particular, in these regions, we found genes that: (i) contain polymorphisms with a high impact or missense effect, (ii) included in list of GIN-activated genes, and (iii) contribute to enrich the most represented GO process in our enrichment analysis. Among these genes, two contributed to enrich the GO terms regulation of immune system process and defense response and mapped to the QTLR region on OAR12: the *TNFRSF1B* (*TNF receptor superfamily member 1B*) gene that harbors a missense mutation (c.103G > A) in exon 2 at position 39,567,687 bp and is very close to the peak of the LDLA analysis (3,943,0517 bp), and the *SELE* (*selectin E*) gene that contains four missense variants. According to the Entrez summary for the human ortholog, *SELE* encodes a protein that is found in cytokine-stimulated endothelial cells and is thought to be responsible for the accumulation of blood leukocytes at sites of inflammation by mediating the adhesion of cells to the vascular lining. In sheep, Gossner et al. [64] found that the *SELE* gene is down-expressed in the abomasal lymph nodes of resistant lambs infected with *T. circumcincta*, which suggests that a possible component of the response of resistant animals to GIN infection could be the repression of acute inflammation and tissues healing.

On OAR19, the most significant peak of the WGS association analysis falls in the first intron of the *GRM7* (*glutamate metabotropic receptor 7*) gene, which is neither included in the list of GIN-activated genes nor contributes to the GO selected terms. However, in the explored QTLR on this chromosome, we found 13 missense variants in the *IL5RA* (*interleukin 5 receptor subunit alpha*) gene, which support the enriched GO term “defense response” in our GO enrichment analysis and appears in the list of GIN-activated genes. Indeed, the *IL5RA* gene was found to have an increased expression in resistant animals in several studies (Scottish Blackface lambs resistant to *T. circumcincta* [64]; Churra resistant sheep infected by the same species [65]; resistant lambs of two different selection flocks of merino sheep [66]).

The QTLR identified on OAR20 is indeed very large and encompasses the MHC region, although the genes from the MHC are located 4 to 6 Mb away from the LDLA most significant location. The MHC complex plays an important role in presenting processed antigens to host T lymphocytes, causing T cell activation and an immunological cascade of events that build the host immunity. Due to the highly polymorphic nature of the MHC region, it is difficult to identify causative mutations useful for selection for GIN resistance [62]. The most significant SNP in the WGS analysis (rs404860665) mapped to the fourth intron of the *LOC101111058* (*butyrophilin-like protein 1*) gene with no function defined in NCBI for sheep. Since no human orthologue of this gene was found in the OrthoDB data base [53], it was not included in the enrichment analysis. However, it is highly expressed in the gastrointestinal tract of sheep (caecum, duodenum, colon, and rectum). Moreover, there is cumulating evidence that butyrophilin-like proteins may have a role as local regulators of intestinal inflammation in other species [67].

In the target region on OAR20, another 20 missense mutations were detected in eight genes (*IL17A*, *IL17F*, *TRIM26*, *TRIM38*, *TNFRSF21*, *LOC101118999*, *VEGFA*, and *TNF*), which are present in the list of GIN-activated genes and contributed to enrich the main GO terms “regulation of immune system process” and “defense response”*.* Among these, the genes encoding interleukins 17 (*IL17A* and *IL17F*), have been mentioned [68] as positional candidates for GIN resistance, but to date, they have not been described in studies on sheep resistance to GIN. However, Gadahi et al. [69] found that IL-17 level was significantly increased in peripheral blood mononuclear cells (PBMC) of goats incubated with *Haemonchus contortus* excretory and secretory proteins (HcESP) and they suggested that such an enhanced IL-17 level might favor the survival of the worm in the host. Moreover, it has been reported that the *IL17F* gene showed the most significant expression difference in the response of the abomasal mucosa of Creole goat kids infected with *Haemonchus contortus*, i.e. its expression was three times higher in resistant compared to susceptible animals [70]. Missense mutations were also detected in the *TNF* (*tumor necrosis factor*) and *TNFRSF21* (*TNF receptor superfamily member 21*) genes. Tumor necrosis factor (TNF) is a cytokine involved in systemic inflammation. The interactions between TNF family ligands and their receptors are involved in the modulation of a number of signaling pathways in the immune system, such as cell proliferation, differentiation, apoptosis and survival [71]. Artis et al. [72] suggested a role for TNF-α in regulating Th2 cytokine responses in the intestine, which has a significant effect on protective immunity to helminth infection. Moreover, the *TNFα* gene was relatively highly expressed in intestinal lymph cells of sheep selected for resistance to nematodes during infection with *Trichostrongylus colubriformis* [73]. In mice, *TNFRSF21-*knockout studies suggest that this gene plays a role in T-helper cell activation, and may be involved in inflammation and immune regulation [71]. A missense mutation was found in the *VEGFA* (*vascular endothelial growth factor A*) gene, which was differentially expressed in abomasal limphonodes of lambs with different susceptibilities to GIN [64] and in the abomasal mucosa of sheep infected with *Haemonchus contortus* [74]. Finally, nine already known missense mutations were detected in the *TRIM26* and *TRIM38* genes. The products of these genes belong to the tripartite motif (TRIM) protein family composed of more than 70 members in humans. Accumulating evidence has indicated that TRIM proteins play crucial roles in the regulation of the pathogenesis of autoimmune diseases and the host defense against pathogens, especially viruses [75]. Both genes were among the GIN-activated genes and contributed to enrich the terms “defense response” (*TRIM38*) and “interferon-gamma-mediated signaling pathway”, “innate immune response”, “defense response” (*TRIM26*). Lyu et al. [76] who investigated the risk associated to nasopharyngeal carcinoma in humans, detected a regulatory variant in this gene and suggested that the downregulation of *TRIM26,* which is dependent on the allele at this variant, contributed to the downregulation of several immune genes and thus was associated to a low immune response.

## Conclusions

Our results show that selective breeding may be an option to limit the issues related to infestation of gastro-intestinal nematodes in sheep. On the one hand, the heritability estimate and QTL detection results confirm that both traditional progeny testing and marker-assisted selection are realistic options. However, the laboriousness of fecal egg counting on a large scale makes marker-assisted selection potentially more profitable in terms of cost benefits. Indeed, the ten significant markers identified in our study and already available on the commercial Illumina arrays explain an important portion of the genetic variation in our large population. On the other hand, the results of the combined use of whole genome data and functional annotation did not provide any marker or causative mutation to improve the efficiency of a marker-assisted selection program in the short term. However, our study which was carried out on a large experimental population provides a first list of candidate genes and SNPs which could be used to address further validation studies on independent populations. In the mid-term, the expected advancements in the quality of the annotation of the ovine genome and the use of experimental designs based on sequence data and phenotypes from multiple breeds that show different LD extents and gametic phases may help to identify causative mutations. As far as the Sarda breed is concerned, the Breeders Association is assessing the feasibility of a selection program for nematode resistance based on fecal egg counting and on the genotypes described in this study for the nucleus flock and combined with the genotyping of selection candidate males that are bred in Herd Book farms and are genetically connected with the experimental flock.

## Supplementary Information

**Additional file 1: Figure S1.** Graphical comparison of LDLA and WGS-based data association analyses within the QTL region Q_01_1 (chromosome 1). The figure shows the test statistics (− log10(nominal p-values) profile of the LDLA analysis (LDLA Mapping, red line) and Manhattan plot of the association analysis based on imputed genotypes from re-sequenced animals (WGS Mapping, blue dots) in the QTL region Q_01_1 (chromosome 1, imputation from 99 to 100 Mb of the *Ovis aries* genome assembly v4.0). **Figure S2.** Graphical comparison of LDLA and WGS-based data association analyses within the QTL region Q_02_1 (chromosome 2). The figure shows the test statistics (− log10(nominal p-values) profile of the LDLA analysis (LDLA Mapping, red line) and Manhattan plot of the association analysis based on imputed genotypes from re-sequenced animals in the QTL region Q_02_1 (chromosome 2, imputation from 135 to 137 Mb of the *Ovis aries* genome assembly v4.0). **Figure S3.** Graphical comparison of LDLA and WGS-based data association analyses within the QTL region Q_02_2 (chromosome 2). The figure shows the test statistics (− log10(nominal p-values) profile of the LDLA analysis (LDLA Mapping, red line) and Manhattan plot of the association analysis based on imputed genotypes from re-sequenced animals in the QTL region Q_02_2 (chromosome 2, imputation from 212 to 214 Mb of the *Ovis aries* genome assembly v4.0). **Figure S4.** Graphical comparison of LDLA and WGS-based data association analyses within the QTL region Q_04_1 (chromosome 4). The figure shows the test statistics (− log10(nominal p-values) profile of the LDLA analysis (LDLA Mapping, red line) and Manhattan plot of the association analysis based on imputed genotypes from re-sequenced animals in the QTL region Q_04_1 (chromosome 4, imputation from 4 to 10 Mb of the *Ovis aries* genome assembly v4.0). **Figure S5.** Graphical comparison of LDLA and WGS-based data association analyses within the QTL region Q_06_1 (chromosome 6). The figure shows the test statistics (− log10(nominal p-values) profile of the LDLA analysis (LDLA Mapping, red line) and Manhattan plot of the association analysis based on imputed genotypes from re-sequenced animals in the QTL region Q_06_1 (chromosome 6, imputation from 12 to 14 Mb of the *Ovis aries* genome assembly v4.0). **Figure S6.** Graphical comparison of LDLA and WGS-based data association analyses within the QTL region Q_07_1 (chromosome 7). The figure shows the test statistics (− log10(nominal p-values) profile of the LDLA analysis (LDLA Mapping, red line) and Manhattan plot of the association analysis based on imputed genotypes from re-sequenced animals in the QTL region Q_07_1 (chromosome 7, imputation from 87 to 89 Mb of the *Ovis aries* genome assembly v4.0). **Figure S7.** Graphical comparison of LDLA and WGS-based data association analyses within the QTL region Q_12_1 (chromosome 12). The figure shows the test statistics (− log10(nominal p-values) profile of the LDLA analysis (LDLA Mapping, red line) and Manhattan plot of the association analysis based on imputed genotypes from re-sequenced animals in the QTL region Q_12_1 (chromosome 7, imputation from 35 to 42 Mb of the *Ovis aries* genome assembly v4.0). **Figure S8.** Graphical comparison of LDLA and WGS-based data association analyses within the QTL region Q_15_1 (chromosome 15). The figure shows the test statistics (− log10(nominal p-values) profile of the LDLA analysis (LDLA Mapping, red line) and Manhattan plot of the association analysis based on imputed genotypes from re-sequenced animals in the QTL region Q_15_1 (chromosome 15, imputation from 33 to 35 Mb of the *Ovis aries* genome assembly v4.0). **Figure S9.** Graphical comparison of LDLA and WGS-based data association analyses within the QTL region Q_19_1 (chromosome 19). The figure shows the test statistics (− log10(nominal p-values) profile of the LDLA analysis (LDLA Mapping, red line) and Manhattan plot of the association analysis based on imputed genotypes from re-sequenced animals in the QTL region Q_19_1 (chromosome 19, imputation from 18 to 32 Mb of the *Ovis aries* genome assembly v4.0). **Figure S10.** Graphical comparison of LDLA and WGS-based data association analyses within the QTL region Q_20_1 (chromosome 20). The figure shows the test statistics (− log10(nominal p-values) profile of the LDLA analysis (LDLA Mapping, red line) and Manhattan plot of the association analysis based on imputed genotypes from re-sequenced animals in the QTL region Q_20_1 (chromosome 20, imputation from 16 to 37 Mb of the *Ovis aries* genome assembly v4.0).

**Additional file 2: Table S1.** Variant classification according to SNPeff 4.3t of bilallelic SNPs identified within the QTL region Q_01_1 on *Ovis aries* chromosome 1. Summary table extracted from the additional snpeff output file”snpEff_summary.html file” reporting the number of effects by impact and the number of effects per type and region, for the QTL region Q_01_1 on chromosome 1 (from 99000291 to 100998839 bp, *Ovis aries* genome assembly v4.0). **Tables S2.** Variant classification according to SNPeff 4.3t of bilallelic SNPs identified within the QTL region Q_02_1 on *Ovis aries* chromosome 2. Summary table extracted from the additional snpeff output file”snpEff_summary.html file” reporting the number of effects by impact and the number of effects per type and region, for the QTL region Q_02_1 on chromosome 2 (from 135000202 to 136999313 bp, *Ovis aries* genome assembly v4.0). **Tables S3.** Variant classification according to SNPeff 4.3t of bilallelic SNPs identified within the QTL region Q_02_2 on *Ovis aries* chromosome 2. Summary table extracted from the additional snpeff output file”snpEff_summary.html file” reporting the number of effects by impact and the number of effects per type and region, for the QTL region Q_02_2 on chromosome 2 (from 212000099 to 213999982 bp, *Ovis aries* genome assembly v4.0). **Tables S4.** Variant classification according to SNPeff 4.3t of bilallelic SNPs identified within the QTL region Q_04_1 on *Ovis aries* chromosome 4. Summary table extracted from the additional snpeff output file”snpEff_summary.html file” reporting the number of effects by impact and the number of effects per type and region, for the QTL region Q_04_1 on chromosome 4 (from 4000037 to 10000000 bp, *Ovis aries* genome assembly v4.0). **Tables S5.** Variant classification according to SNPeff 4.3t of bilallelic SNPs identified within the QTL region Q_06_1 on *Ovis aries* chromosome 6. Summary table extracted from the additional snpeff output file”snpEff_summary.html file” reporting the number of effects by impact and the number of effects per type and region, for the QTL region Q_06_1 on chromosome 6 (from 12000078 to 13999887 bp, *Ovis aries* genome assembly v4.0). **Tables S6.** Variant classification according to SNPeff 4.3t of bilallelic SNPs identified within the QTL region Q_07_1 on *Ovis aries* chromosome 7. Summary table extracted from the additional snpeff output file”snpEff_summary.html file” reporting the number of effects by impact and the number of effects per type and region, for the QTL region Q_07_1 on chromosome 7 (from 87000021 to 88999946 bp, *Ovis aries* genome assembly v4.0). **Tables S7**. Variant classification according to SNPeff 4.3t of bilallelic SNPs identified within the QTL region Q_12_1 on *Ovis aries* chromosome 12. Summary table extracted from the additional snpeff output file”snpEff_summary.html file” reporting the number of effects by impact and the number of effects per type and region, for the QTL region Q_12_1 on chromosome 12 (from 35000043 to 41999843 bp, *Ovis aries* genome assembly v4.0). **Tables S8.** Variant classification according to SNPeff 4.3t of bilallelic SNPs identified within the QTL region Q_15_1 on *Ovis aries* chromosome 15. Summary table extracted from the additional snpeff output file”snpEff_summary.html file” reporting the number of effects by impact and the number of effects per type and region, for the QTL region Q_15_1 on chromosome 15 (from 33000037 to 34999984 bp, *Ovis aries* genome assembly v4.0). **Tables S9.** Variant classification according to SNPeff 4.3t of bilallelic SNPs identified within the QTL region Q_19_1 on *Ovis aries* chromosome 19. Summary table extracted from the additional snpeff output file”snpEff_summary.html file” reporting the number of effects by impact and the number of effects per type and region, for the QTL region Q_19_1 on chromosome 19 (from 18000014 to 31999894 bp, *Ovis aries* genome assembly v4.0). **Tables S10.** Variant classification according to SNPeff 4.3t of bilallelic SNPs identified within the QTL region Q_20_1 on *Ovis aries* chromosome 20. Summary table extracted from the additional snpeff output file”snpEff_summary.html file” reporting the number of effects by impact and the number of effects per type and region, for the QTL region Q_20_1 on chromosome 20 (from 16000304 to 36997864 bp, *Ovis aries* genome assembly v4.0).

**Additional file 3: Table S11.** Full characterisation of the retained SNPs: high or moderate impact variants or most significant variants from the association analysis mapping within the QTL regions. Description of the retained SNPs that mapped within the QTL regions identified in the present work: functional annotation from SNPeff; nominal significance level (− log10(nominal p-values) from the WGS based association analysis, GO biological process enriched term from WebGestalt analysis; and study from which the candidate GIN-activated gene listed by Chitneedi et al. 2020 [51] was identified. The SNP positions are from the *Ovis aries* genome assembly v4.0.

**Additional file 4: Table S12.** Functional characterization of the 10 most significant SNPs per QTLR from the WGS analysis. Characterization of the 10 most significant SNPs of the QTLR considered in this work and their functional consequences according to the annotation performed with SnpEff. The SNP positions are from the *Ovis aries* genome assembly v4.0.

**Additional file 5: Table S13.** Top hierarchical terms identified by the Gene Ontology (GO) enrichment analysis (biological process database) performed with WebGestalt. Results of the over-representation analysis (ORA) of GO biological process terms of the genes harboring significant mutations or mutations with functional consequences on the transcripts performed with WebGestalt. Gene symbols and ID of human gene orthologues are reported. They were retrieved from the OrthoDB v10 data base starting from the NBCI ID of ovine genes from the *Ovis aries* annotation release 102.

## Data Availability

The data that support the findings of this study are available from Centro Regionale di Programmazione (CRP), Regione Autonoma della Sardegna but restrictions apply to the availability of these data, which were used under license for the current study, and thus are not publicly available. However, data are available from the authors upon reasonable request and with permission of Centro Regionale di Programmazione (CRP), Regione Autonoma della Sardegna.
