# Supplementary figures and images for: Association analysis and functional annotation of imputed sequence data within genomic regions influencing resistance to gastro-intestinal parasites detected by an LDLA approach in a nucleus flock of Sarda dairy sheep
Source: Genet Sel Evol. 2022 Jan 3;54:2. doi: 10.1186/s12711-021-00690-7 (PMC8722200; doi:10.1186/s12711-021-00690-7)

Figure S


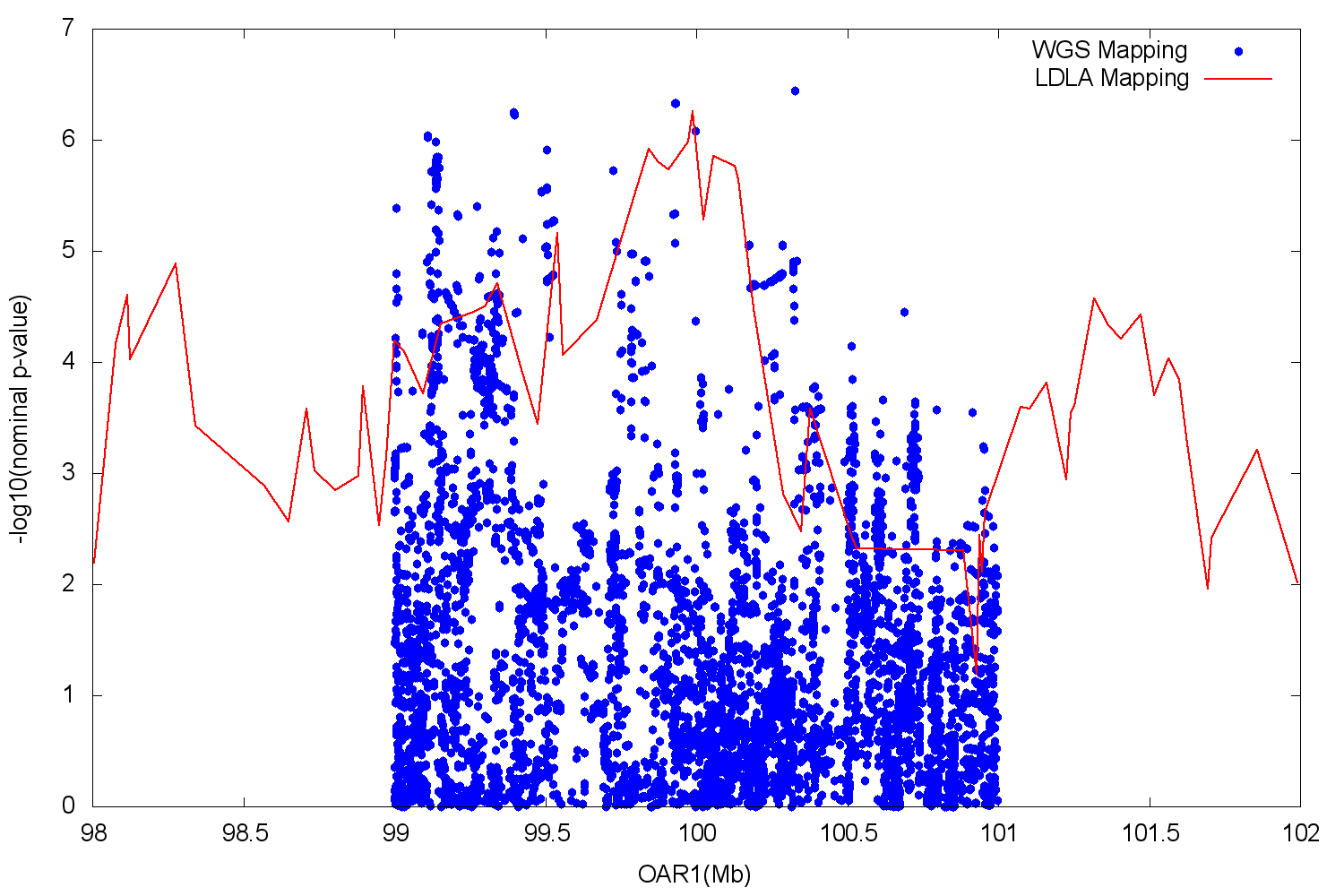


Figure S


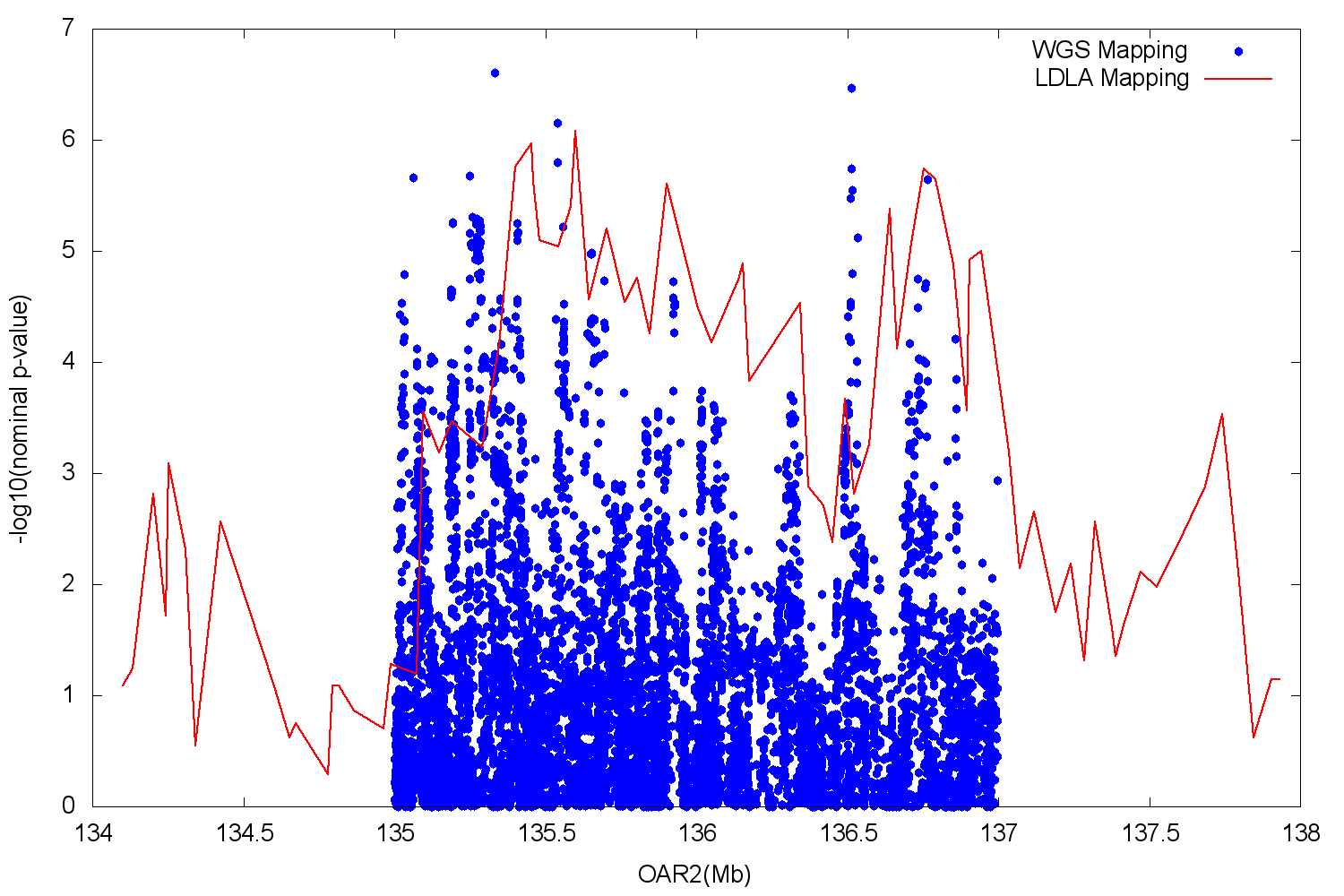


Figure S


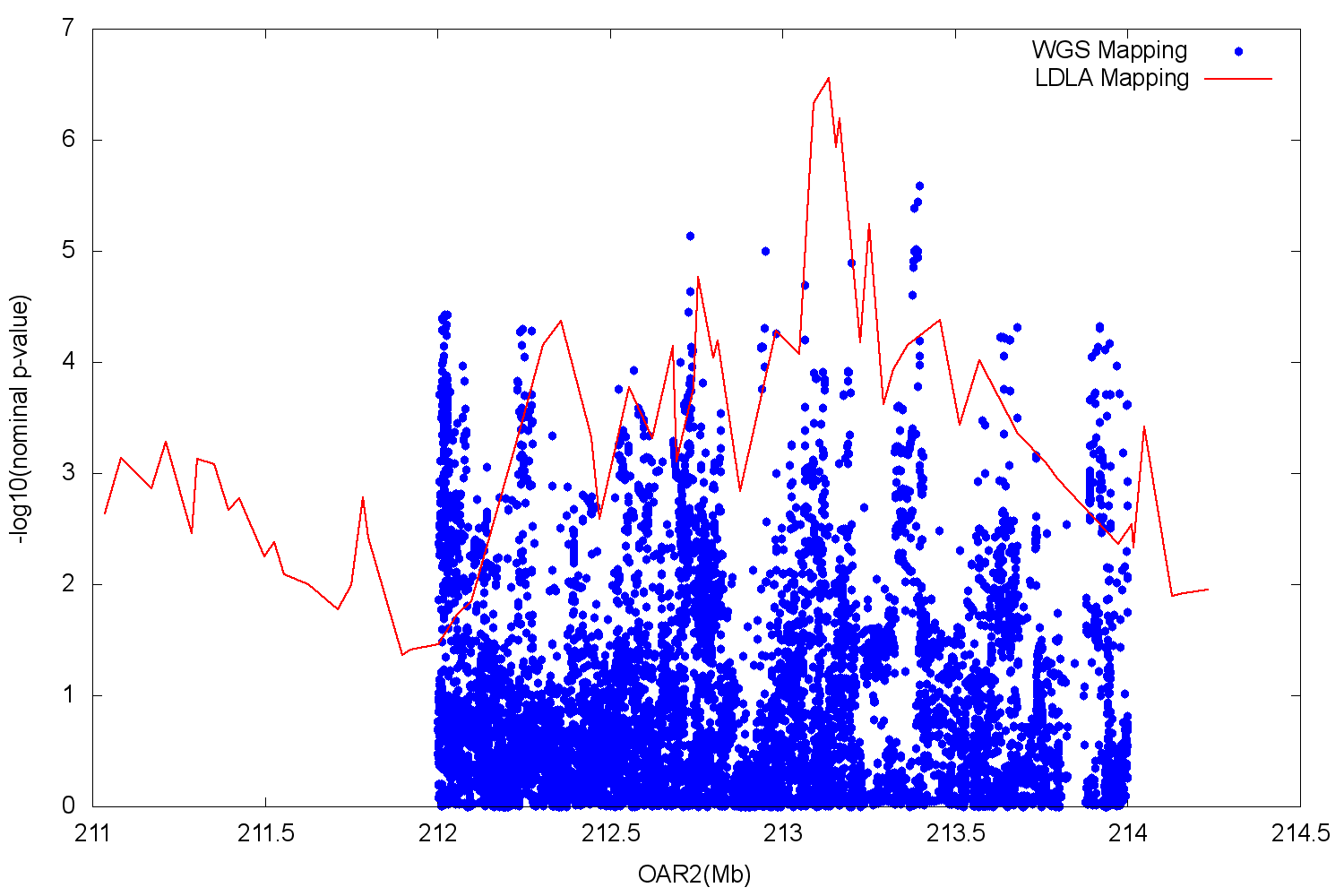


Figure S


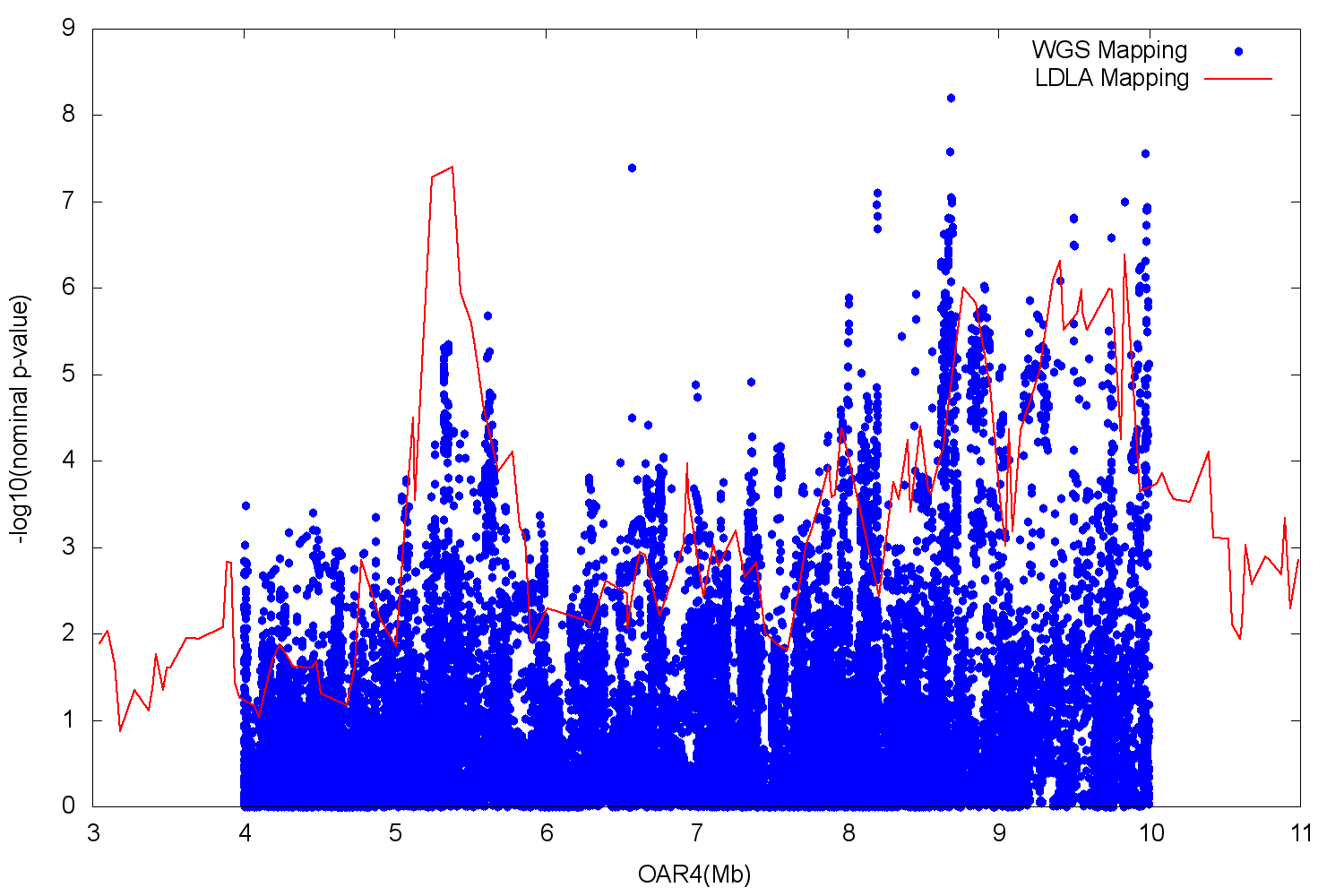


Figure S


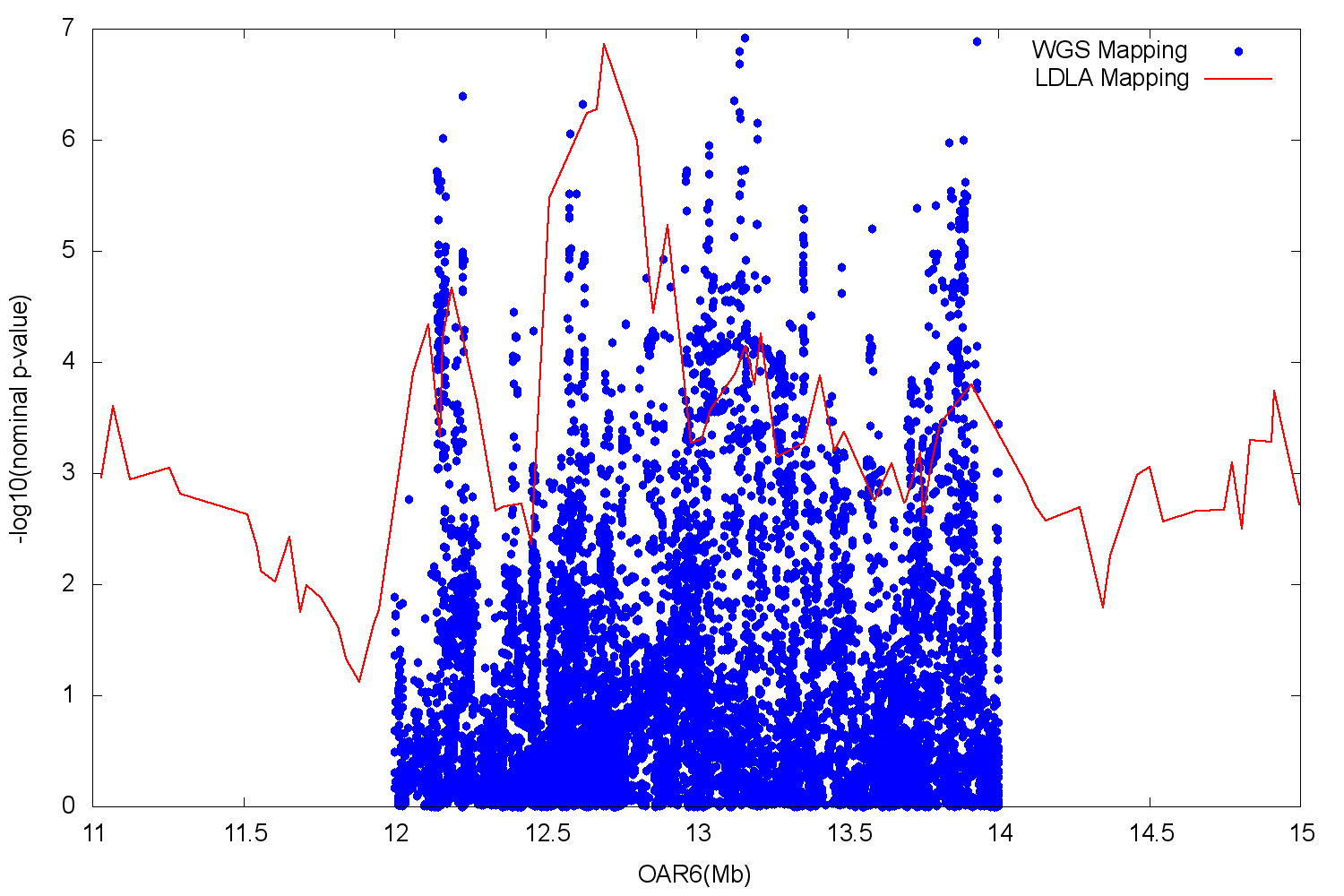


Figure S


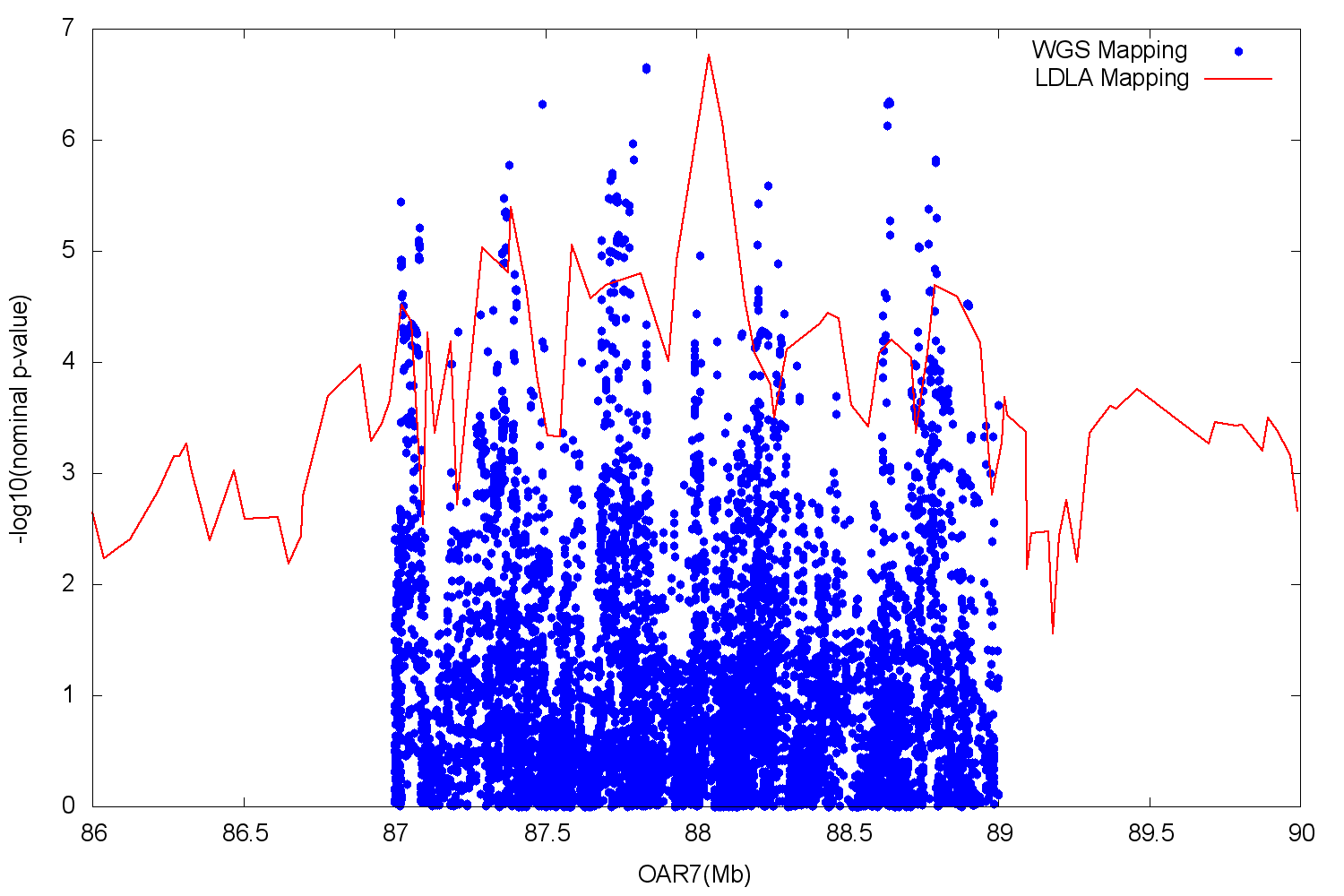


Figure S


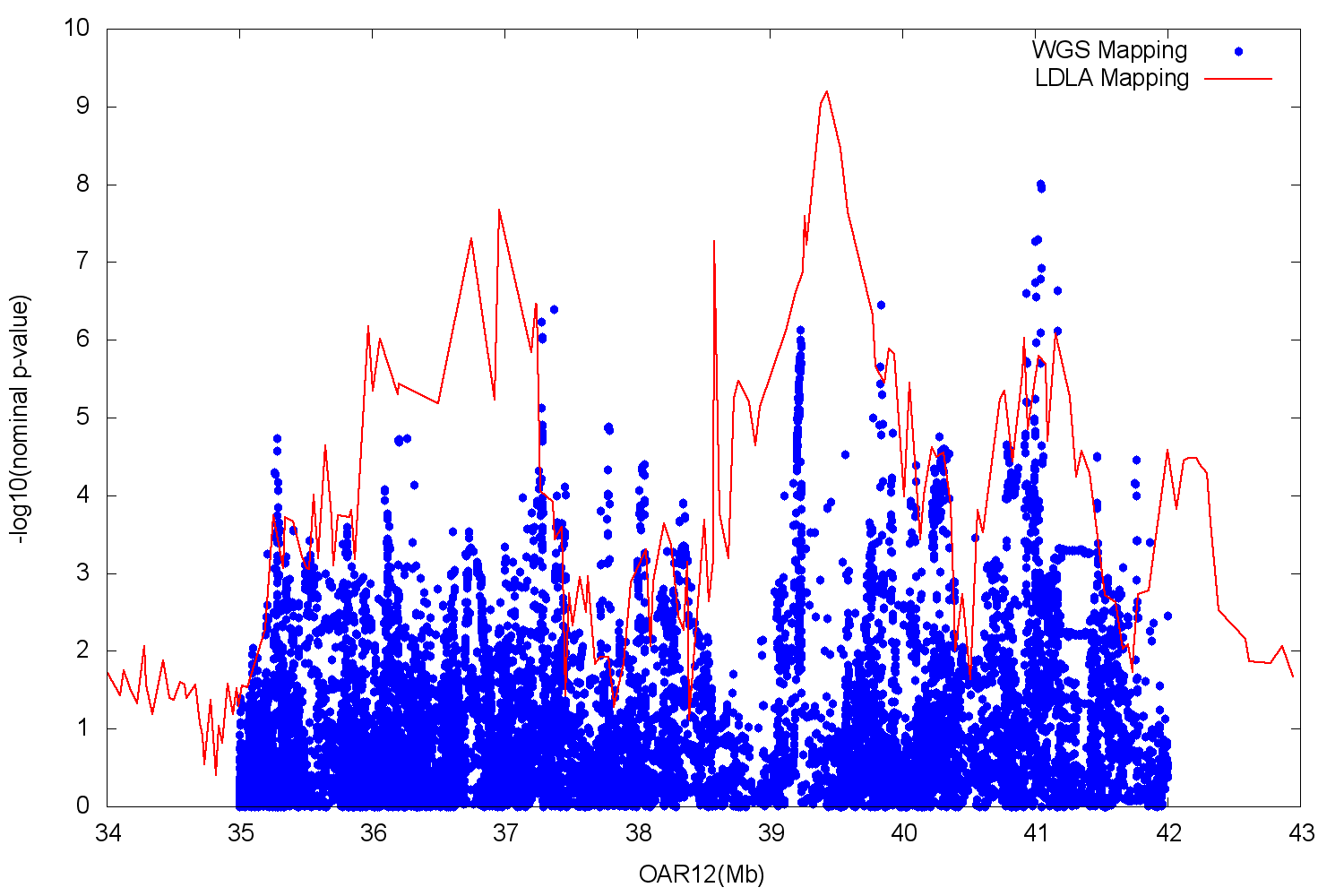


Figure S


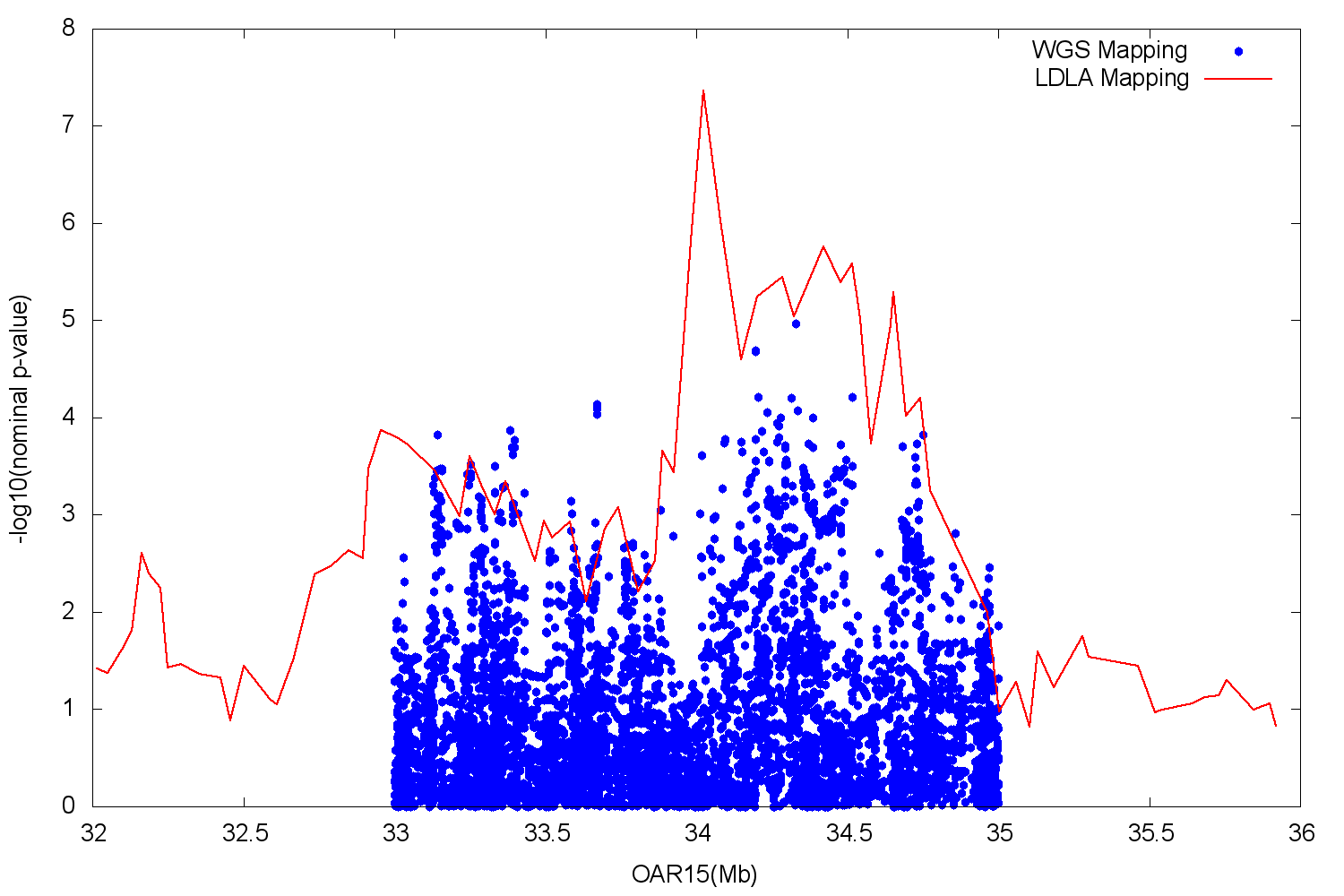


Figure S


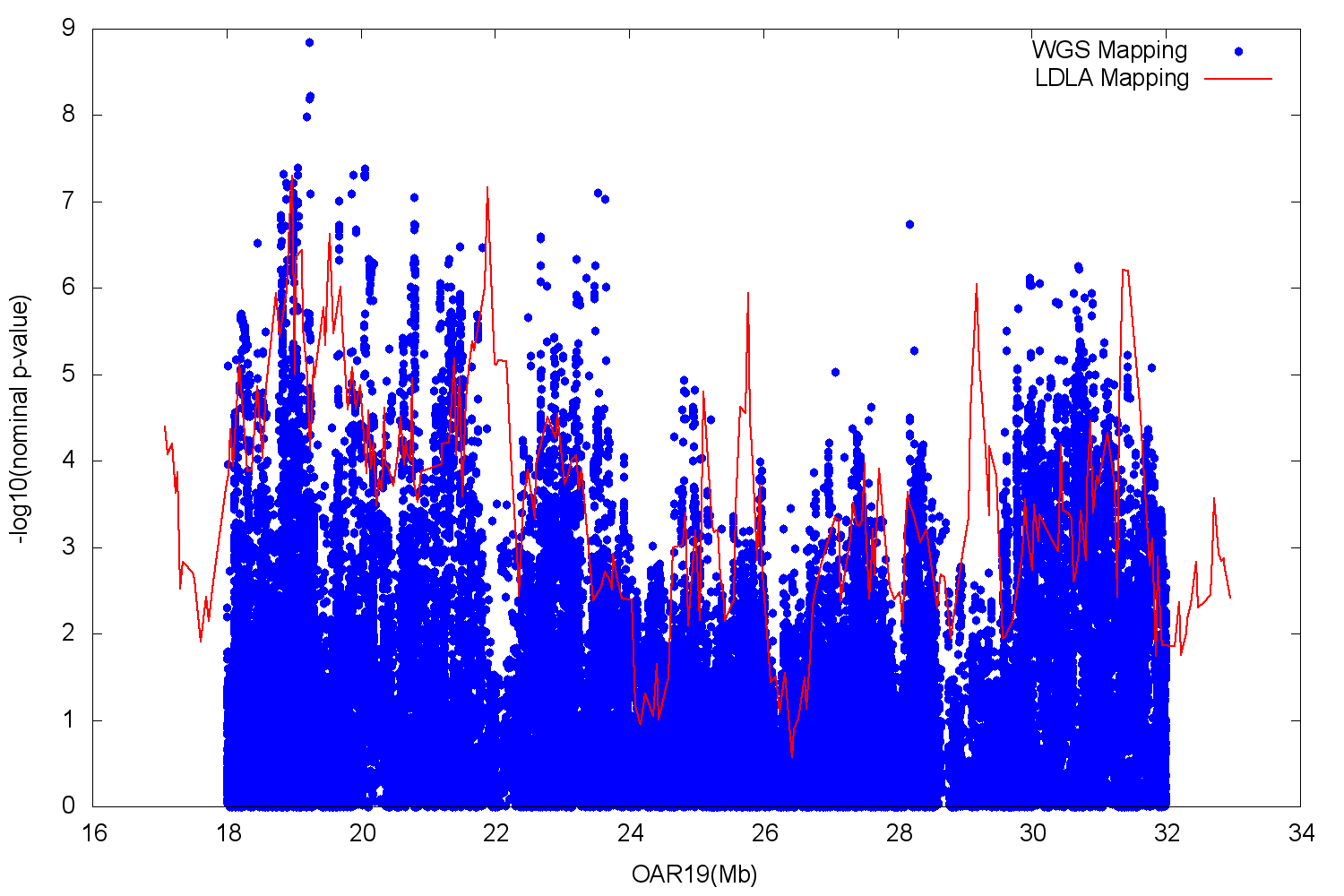


Figure S


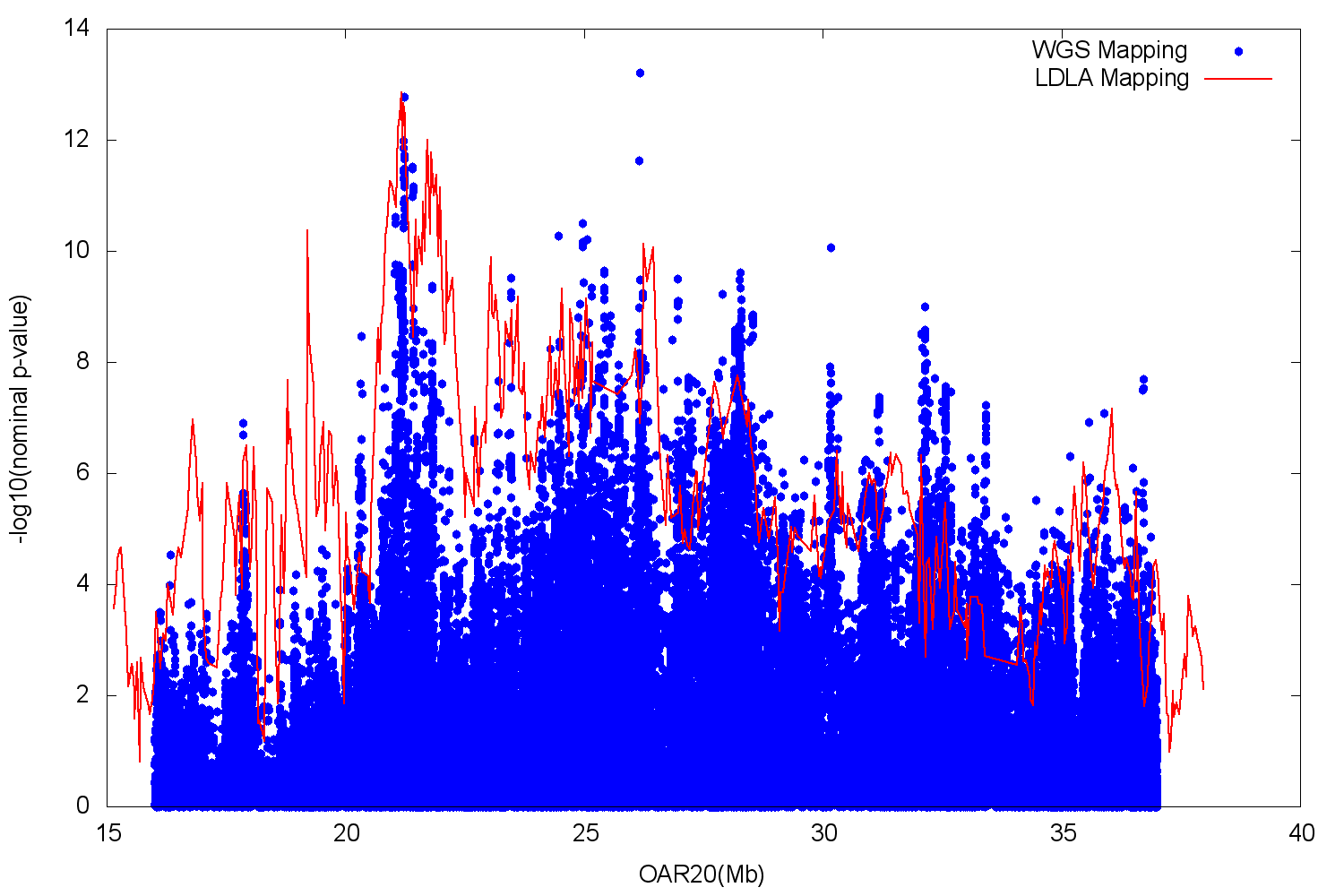

Supplement: Supplementary file 1 — Additional file 1: Figure S1. Graphical comparison of LDLA and WGS-based data association analyses within the QTL region Q_01_1 (chromosome 1). The figure shows the test statistics (− log10(nominal p-values) profile of the LDLA analysis (LDLA Mapping, red line) and Manhattan plot of the association analysis based on imputed genotypes from re-sequenced animals (WGS Mapping, blue dots) in the QTL region Q_01_1 (chromosome 1, imputation from 99 to 100 Mb of the Ovis aries genome assembly v4.0). Figure S2. Graphical comparison of LDLA and WGS-based data association analyses within the QTL region Q_02_1 (chromosome 2). The figure shows the test statistics (− log10(nominal p-values) profile of the LDLA analysis (LDLA Mapping, red line) and Manhattan plot of the association analysis based on imputed genotypes from re-sequenced animals in the QTL region Q_02_1 (chromosome 2, imputation from 135 to 137 Mb of the Ovis aries genome assembly v4.0). Figure S3. Graphical comparison of LDLA and WGS-based data association analyses within the QTL region Q_02_2 (chromosome 2). The figure shows the test statistics (− log10(nominal p-values) profile of the LDLA analysis (LDLA Mapping, red line) and Manhattan plot of the association analysis based on imputed genotypes from re-sequenced animals in the QTL region Q_02_2 (chromosome 2, imputation from 212 to 214 Mb of the Ovis aries genome assembly v4.0). Figure S4. Graphical comparison of LDLA and WGS-based data association analyses within the QTL region Q_04_1 (chromosome 4). The figure shows the test statistics (− log10(nominal p-values) profile of the LDLA analysis (LDLA Mapping, red line) and Manhattan plot of the association analysis based on imputed genotypes from re-sequenced animals in the QTL region Q_04_1 (chromosome 4, imputation from 4 to 10 Mb of the Ovis aries genome assembly v4.0). Figure S5. Graphical comparison of LDLA and WGS-based data association analyses within the QTL region Q_06_1 (chromosome 6). The figure shows th [file 12711_2021_690_MOESM1_ESM.docx]
